# Resolving sepsis-induced immunoparalysis via trained immunity by targeting interleukin-4 to myeloid cells

**DOI:** 10.1038/s41551-023-01050-0

**Published:** 2023-06-08

**Authors:** David P. Schrijver, Rutger J. Röring, Jeroen Deckers, Anne de Dreu, Yohana C. Toner, Geoffrey Prevot, Bram Priem, Jazz Munitz, Eveline G. Nugraha, Yuri van Elsas, Anthony Azzun, Tom Anbergen, Laszlo A. Groh, Anouk M. D. Becker, Carlos Pérez-Medina, Roderick S. Oosterwijk, Boris Novakovic, Simone J. C. F. M. Moorlag, Aron Jansen, Peter Pickkers, Matthijs Kox, Thijs J. Beldman, Ewelina Kluza, Mandy M. T. van Leent, Abraham J. P. Teunissen, Roy van der Meel, Zahi A. Fayad, Leo A. B. Joosten, Edward A. Fisher, Maarten Merkx, Mihai G. Netea, Willem J. M. Mulder

**Affiliations:** 1 Department of Biomedical Engineering, Eindhoven University of Technology, Eindhoven, the Netherlands; 2 Department of Internal Medicine and Radboud Center for Infectious Diseases (RCI), Radboud University Medical Center, Nijmegen, the Netherlands; 3 Radboud Institute for Molecular Life Sciences, Radboud University Medical Center, Nijmegen, the Netherlands; 4 Biomedical Engineering and Imaging Institute, Icahn School of Medicine at Mount Sinai, New York, NY USA; 5 Department of Medical Biochemistry, Amsterdam University Medical Centers, Amsterdam, the Netherlands; 6 Angiogenesis Laboratory, Amsterdam UMC, Cancer Center Amsterdam, Amsterdam, the Netherlands; 7 Department of Surgery, Radboud University Medical Center, Nijmegen, the Netherlands; 8 Radboud Institute for Health Sciences, Radboud University Medical Center, Nijmegen, the Netherlands; 9 Department of Tumor Immunology, RIMLS, Radboud University Medical Center, Nijmegen, the Netherlands; 10 Centro Nacional de Investigaciones Cardiovasculares (CNIC), Madrid, Spain; 11 Epigenetics Group, Murdoch Children’s Research Institute, Royal Children’s Hospital and Department of Paediatrics, University of Melbourne, Parkville, Victoria Australia; 12 Department of Intensive Care Medicine and Radboud Center for Infectious Diseases (RCI), Radboud University Medical Center, Nijmegen, the Netherlands; 13 Cardiovascular Research Institute, Icahn School of Medicine at Mount Sinai, New York, NY USA; 14 Department of Medical Genetics, Iuliu Hațieganu University of Medicine and Pharmacy, Cluj-Napoca, Romania; 15 Division of Cardiology, Department of Medicine, Marc and Ruti Bell Program in Vascular Biology, New York University School of Medicine, New York, NY USA; 16 Department for Genomics & Immunoregulation, Life and Medical Sciences Institute (LIMES), University of Bonn, Bonn, Germany

**Keywords:** Interleukins, Monocytes and macrophages, Protein delivery

## Abstract

Immunoparalysis is a compensatory and persistent anti-inflammatory response to trauma, sepsis or another serious insult, which increases the risk of opportunistic infections, morbidity and mortality. Here, we show that in cultured primary human monocytes, interleukin-4 (IL4) inhibits acute inflammation, while simultaneously inducing a long-lasting innate immune memory named trained immunity. To take advantage of this paradoxical IL4 feature in vivo, we developed a fusion protein of apolipoprotein A1 (apoA1) and IL4, which integrates into a lipid nanoparticle. In mice and non-human primates, an intravenously injected apoA1-IL4-embedding nanoparticle targets myeloid-cell-rich haematopoietic organs, in particular, the spleen and bone marrow. We subsequently demonstrate that IL4 nanotherapy resolved immunoparalysis in mice with lipopolysaccharide-induced hyperinflammation, as well as in ex vivo human sepsis models and in experimental endotoxemia. Our findings support the translational development of nanoparticle formulations of apoA1-IL4 for the treatment of patients with sepsis at risk of immunoparalysis-induced complications.

## Abstract (web summary)

A nanoparticle integrating a fusion protein of apolipoprotein A1 and interleukin-4 and that targets myeloid-cell-rich haematopoietic organs resolves immunoparalysis in ex vivo and in vivo models of sepsis.

## Main

Sepsis is a serious medical condition caused by a dysregulated host response to infection that frequently results in organ failure and death^1^. As a result of the immune system’s inability to clear a pathogen, patients with sepsis may experience simultaneous hyper-inflammatory and immunosuppressive characteristics, making this condition extremely challenging to manage^2–4^. Up to 30–40% of patients with sepsis show an overriding immunoparalysis phenotype. Immunoparalysis is characterized by a persistent anti-inflammatory innate immune response following an insult such as sepsis^5^, putting patients at high risk of recurrent and secondary infections, frequently leading to organ dysfunction and death^6^.

Therapeutic strategies to rebalance immune responses in sepsis and to improve patient outcomes through immunotherapy are in their infancy. Recent experimental work suggests that the long-term reprogramming of the innate immune cells^7,8^, a process termed ‘trained immunity’^9,10^, may be able to reverse tolerance and immunoparalysis induced by exaggerated bacterial stimulation^11^. Although therapeutically induced trained immunity^12^ is theoretically a compelling strategy to overcome immunoparalysis, a critical need exists for approaches that can be safely and efficiently translated to clinical settings.

In our quest to simultaneously resolve hyper-inflammation and immunoparalysis, we considered interleukin-4 (IL4) for the modulation of trained immunity and tolerance. When re-evaluating the reported direct effects of this cytokine on monocytes in vitro^13–15^, we discovered that IL4 paradoxically induces trained immunity in addition to its known anti-inflammatory effects. We hypothesized that the unique properties of IL4 could be deployed to overcome immunoparalysis in monocytes induced by stimulation with bacterial endotoxin (lipopolysaccharide (LPS)). However, owing to its unfavourable pharmacokinetic properties and the broad IL4 receptor (IL4R) expression, natural IL4 is poorly suitable for therapeutically regulating myeloid cells in vivo. Routing IL4 directly to the myeloid compartment is therefore an attractive therapeutic avenue. In support of this concept, we developed a fusion protein of apolipoprotein A1 (apoA1)—the main protein constituent of high-density lipoprotein—and IL4 and termed this construct ‘apoA1–IL4’^16,17^. The apoA1–IL4 fusion protein readily integrated into lipid nanoparticles to generate myeloid cell-avid IL4 nanoparticles. We then evaluated the behaviour of the IL4 nanoparticles in mice and non-human primates using in vivo positron emission tomography (PET) imaging and ex vivo quantitative gamma counting. Finally, we studied the therapeutic potential of the IL4 nanoparticles in multiple translational inflammation and in vivo and ex vivo models of sepsis.

## Results

### IL4 inhibits acute inflammation, yet induces trained immunity

**Fig. 1:**
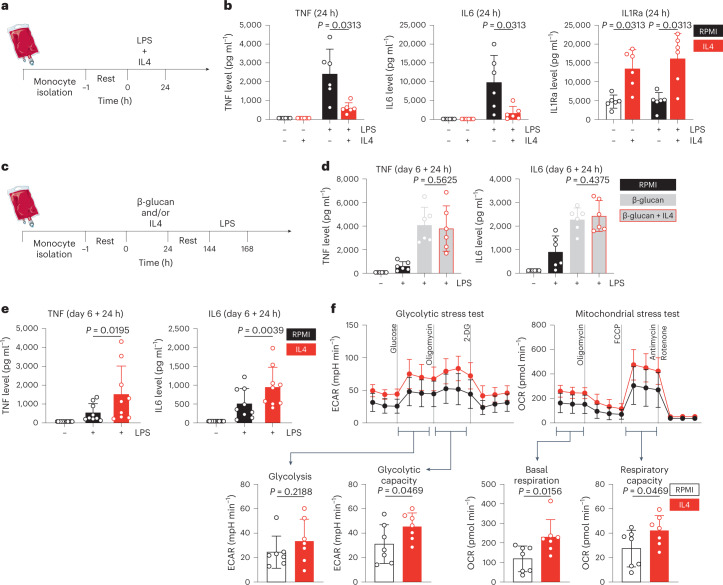
IL4 inhibits acute inflammation, yet induces trained immunity. **a**, Schematic of in vitro direct inflammation experiments. **b**, TNF, IL6 and IL1Ra levels after 24 h stimulation of human primary monocytes. **c**, Schematic of in vitro trained immunity experiments. **d**, TNF and IL6 levels after re-stimulation of β-glucan-trained cells. **e**, TNF and IL6 levels after re-stimulation of IL4-trained cells. **f**, Seahorse analysis of glycolytic (left) and mitochondrial (right) metabolism in IL4-trained cells. Data are presented as mean ± s.d. OCR, oxygen consumption rate; 2-DG, 2-deoxy-D-glucose.

**Extended Data Fig. 1:**
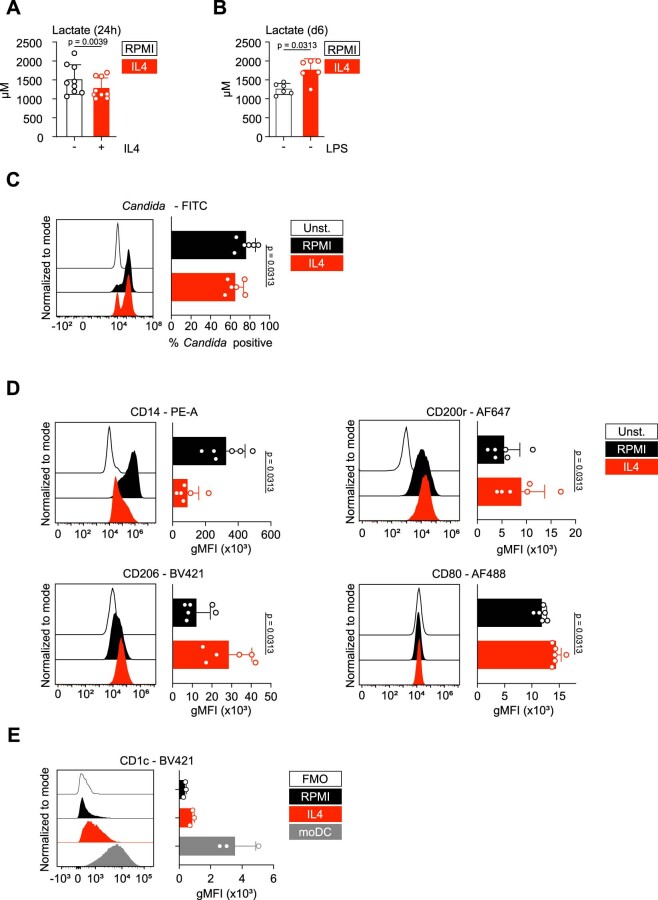
IL4 inhibits acute inflammation, yet induces trained immunity. **(a**) Supernatant lactate levels after 24-hour stimulation with IL4. (**b**) Accumulated supernatant lactate levels on day 6 after IL4 training. (**c**) Flow cytometric measurement of FITC-labelled *Candida albicans* phagocytosis on day 6 after IL4-training. (**d**) Flow cytometric measurement of surface markers commonly associated with IL4 activation of monocytes/macrophages, on day 6 after IL4-training. (**e**) Flow cytometric measurement of the dendritic cell marker CD1c on IL4-trained macrophages and monocyte-derived dendritic cells (moDC). Data are presented as mean (flow cytometry: geometric mean fluorescence intensity) ± SD.

In the context of myeloid cell immunology, IL4 is known primarily for its anti-inflammatory properties^13–15^. Therefore, we first validated several known inhibitory effects of IL4 on inflammation in primary human monocytes (Fig. 1a). We stimulated Percoll-enriched monocytes with LPS for 24 h, in the presence or absence of IL4 (25 ng ml^−1^). As expected, IL4 potently inhibited the secretion of the pro-inflammatory cytokines tumour necrosis factor (TNF) and IL6 (Fig. 1b). Interestingly, IL4-treated cells secreted significantly more IL-1Ra compared to controls (Fig. 1b). As glycolysis is upregulated in activated myeloid cells^18^, we measured lactate production in otherwise unstimulated monocytes treated with IL4 or a medium control. We found IL4 to slightly, but significantly, lower baseline lactate production (Extended Data Fig. 1a), confirming its acute anti-inflammatory properties.

On the basis of these anti-inflammatory properties, we hypothesized that IL4 might also inhibit the induction of trained immunity (Fig. 1c). To test this hypothesis, monocytes were trained with β-glucan, a prototypical trained immunity stimulus, for 24 h, followed by washing away the stimulus and a 5 day resting period in culture medium. On day 6, we re-stimulated the cells with LPS for another 24 h and measured TNF and IL6 (Fig. 1d). While β-glucan induced trained immunity as expected, addition of IL4 in the first 24 h did not inhibit the training effect (Fig. 1d). Contrary to our initial hypothesis, exposing monocytes to IL4 alone for 24 h induced a trained immunity phenotype on day 6 (Fig. 1e). Besides enhanced production of pro-inflammatory cytokines, IL4-trained cells produced more lactate at baseline (Extended Data Fig. 1b). Furthermore, IL4-trained cells were slightly less effective at phagocytosing heat-killed *Candida albicans* than untrained controls (Extended Data Fig. 1c). Collectively, our data show that IL4 inhibits inflammation and induces trained immunity, at both metabolic and functional immunologic levels.

Encouraged by these observations, we comprehensively studied the metabolic alterations following IL4-induced trained immunity. To this aim, we used Seahorse metabolic flux analyses to probe glycolytic and oxidative metabolism of IL4-trained cells and unstimulated controls. IL4 training on day 0 had a marked effect on metabolic parameters measured on day 6 (Fig. 1f), with a trend towards higher basal glycolysis and a significant increase of oligomycin-triggered maximum glycolytic capacity (Fig. 1f, left). In addition, both baseline- and carbonyl cyanide-*p*-trifluoromethoxyphenylhydrazone-triggered maximum respiration rates were significantly augmented by IL4 training (Fig. 1f; right).

We then used flow cytometry to measure several parameters commonly associated with IL4 activation of monocytes and macrophages (Extended Data Fig. 1d). IL4 training caused a strong downregulation of cluster of differentiation (CD)14 expression on day 6. In contrast, CD200R and especially CD206 were significantly enhanced on day 6 subsequent to IL4 activation on day 0. CD80 was marginally increased by IL4 training, but overall expression was still low on these otherwise naive macrophages. It is known that monocyte-derived dendritic cells (moDCs, which are differentiated using IL4+granulocyte-macrophage colony-stimulating factor (GM-CSF)) also downregulate CD14 while strongly upregulating CD1c. IL4-trained cells expressed slightly more CD1c than untrained cells but far less than moDCs (Extended Data Fig. 1e). These results indicate IL4 induces a program of trained immunity that incorporates features known from classical IL4 immunological functions.

### Immune and epigenetic mechanisms mediating IL4-induced trained immunity

**Fig. 2:**
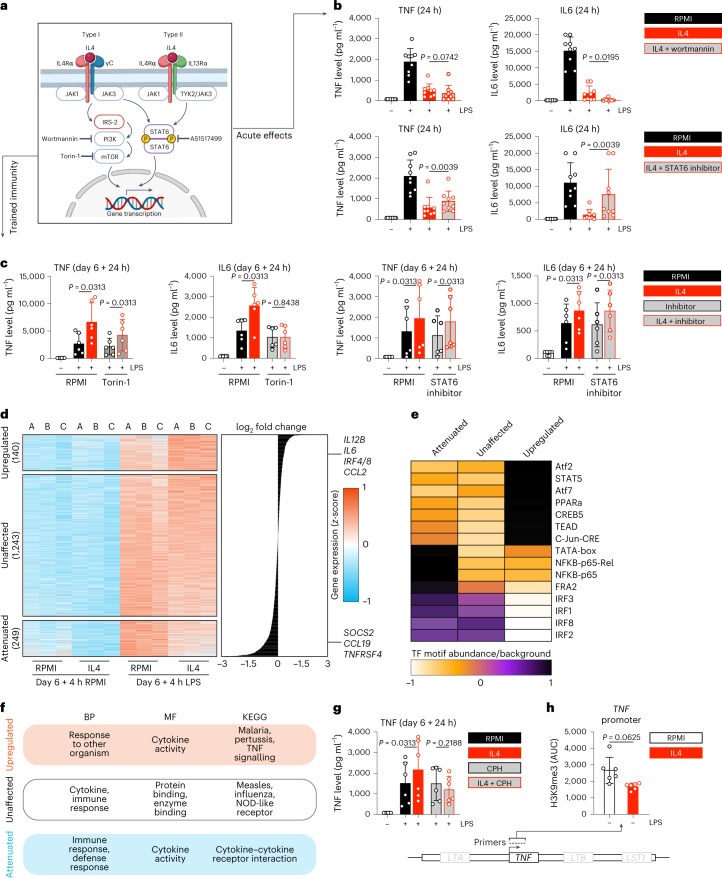
Immune and epigenetic mechanisms mediating IL4-induced trained immunity. **a**, Schematic overview of previously described premier IL4 signalling pathways^19^. Generated using Biorender. **b**, TNF and IL6 levels after 24 h stimulation of monocytes while blocking key IL4 signalling routes. **c**, TNF and IL6 levels after re-stimulation of cells that were trained with IL4 while blocking key IL4 signalling routes. **d**, Heat map of the transcriptome of IL4-trained cells, before and after re-stimulation. **e**, TF motif enrichment analysis in IL4-trained immunity (heat map indicates *z-*scores). **f**, Pathway enrichment analyses of the IL4-trained immunity transcriptome. **g**, TNF and IL6 levels after re-stimulation of cells that were trained with IL4 in the presence of a SET7 methyltransferase inhibitor. CPH, cyproheptadine. **h**, ChIP-qPCR AUC analysis of *TNF* in IL4-trained cells. Data in bar graphs are presented as mean ± s.d.

**Extended Data Fig. 2:**
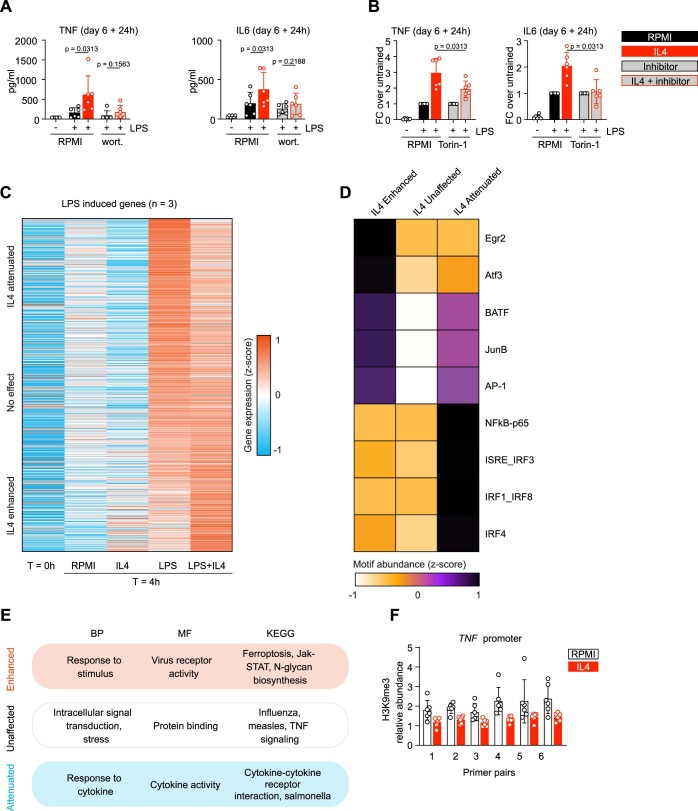
Immune and epigenetic mechanisms mediating IL4-induced trained immunity. (**a**) TNF/IL6 production after re-stimulation of cells that were trained with IL4 whilst blocking key IL4 signaling routes (**b**) Fold increase of TNF/IL6 after re-stimulation of cells that were trained with IL4 whilst blocking key IL4 signaling routes. (**c**) Heatmap of the transcriptome of monocytes directly after isolation, and after stimulation with RPMI, IL4, LPS, or LPS+IL4. (**d**) Transcription factor motif enrichment analysis of acute IL4 effects on LPS-stimulated monocytes. (**e**) Pathway enrichment analyses of IL4’s effect on acute LPS stimulation. (**f**) ChIP-qPCR analysis of *TNF* in IL4-trained cells. Data are presented as mean ± SD.

The signalling mechanisms of IL4 are well described: the insulin receptor substrate 2-phosphoinositide 3-kinases-mammalian target of rapamycin (IRS-2–PI3K–mTOR) axis and the signal transducer and activator of transcription 6 (STAT6) signalling pathway^19^ (Fig. 2a). We performed pharmacological inhibition experiments to investigate the role of these pathways for both inhibition of acute inflammation and trained immunity induction by IL4. Inhibition of PI3K or mTOR (using wortmannin or torin-1, respectively) did not abrogate the effect of IL4 on acute inflammation but diminished the trained immunity responses (Fig. 2b,c and Extended Data Fig. 2a). IL4 training, as measured by an increased TNF and IL6 production, was significantly blunted in the presence of torin-1 (Fig. 2c and Extended Data Fig. 2b). In contrast, the STAT6 inhibitor AS1517499 partly restored cytokine production in acute inflammatory responses but did not affect trained immunity induction by IL4 (Fig. 2b,c). Thus, each of the signalling pathways induced downstream of IL4 engagement with its receptors has distinct functions: IL4 exerts its known acute anti-inflammatory function through STAT6 but simultaneously induces trained immunity via PI3K–mTOR, a previously unknown pro-inflammatory effect.

To gain insight into the molecular program induced by IL4 training, we performed transcriptomics analysis on naive and IL4-trained macrophages, both before and after LPS re-stimulation on day 6. Overall, 140 genes were more strongly induced (‘upregulated’) in IL4-trained macrophages, whereas 249 genes were attenuated (Fig. 2d). Among the top upregulated genes were pro-inflammatory cytokines such as *IL6* and *IL12B* that are known to be involved in trained immunity^10^. Among the prominent attenuated genes were *CCL19* and *SOCS2*, which are important for lymphocyte trafficking and suppression of cytokine signalling, respectively^20,21^.

We next performed transcription factor (TF) motif enrichment analysis (Fig. 2e) and gene ontology and pathway enrichment analyses (Fig. 2f) to gain further insight into the transcriptome profiles. Promoters of genes upregulated in IL4-trained macrophages were highly enriched for motifs recognized by TFs such as activating transcription factor (ATF)2/ATF7, peroxisome proliferator-activated receptor alpha (PPARα) and STAT5, whereas interferon regulatory factor (IRF) motifs were especially depleted. This pattern was mostly reversed for unaffected and attenuated genes, except for TATA-box, nuclear factor kappa-light-chain-enhancer of activated B cells (NFκB)-p65, NFκB-p65-Rel and fos-related antigen 2: these motifs were highly enriched in attenuated gene promoters but decreased in both unaffected and upregulated genes (Fig. 2e). Gene ontology (biological process (BP) and molecular function (MF)) and Kyoto Encyclopedia of Genes and Genomes pathway enrichment showed that immunological activities were present in both upregulated (for example, BP ‘response to organism’, Kyoto Encyclopedia of Genes and Genomes ‘TNF signalling’) and attenuated (for example, BP ‘immune response’, MF ‘cytokine activity’) gene sets (Fig. 2f). We performed a similar transcriptome analysis on monocytes stimulated immediately after isolation with IL4, LPS or IL4 and LPS combined, which confirmed an acute anti-inflammatory transcriptomic response to IL4 (Extended Data Fig. 2c–e). Together, these data reveal specific transcriptional programs in both the acute anti-inflammatory effects and the long-term trained immunity responses invoked by IL4.

We subsequently investigated the importance and presence of epigenetic reprogramming, specifically histone modifications. Addition of the anti-allergy drug cyproheptadine, a su(var)3-9, enhancer-of-zeste and trithorax-domain containing lysine methyltransferase 7 (SET7) (also known as SET9) histone methyltransferase inhibitor, abrogated the induction of trained immunity by IL4 (Fig. 2g). SET7 has been described earlier as an important epigenetic mediator of trained immunity^22^. Furthermore, we evaluated histone-3-lysine-9-tri-methylation (H3K9me3)-mediated repression of *TNF* using chromatin immunoprecipitation (ChIP)–quantitative PCR (qPCR) analyses in IL4-induced trained immunity. Using an area under the curve (AUC) analysis of six primer pairs showed a decrease of H3K9me3 in IL4-induced trained immunity, although this did not reach statistical significance (Fig. 2h and Extended Data Fig. 2f). Together, these data indicate epigenetic reprogramming is crucial for, and characteristic of, IL4-induced trained immunity.

### Developing an apoA1–IL4 fusion protein that integrates in lipid nanoparticles

**Fig. 3:**
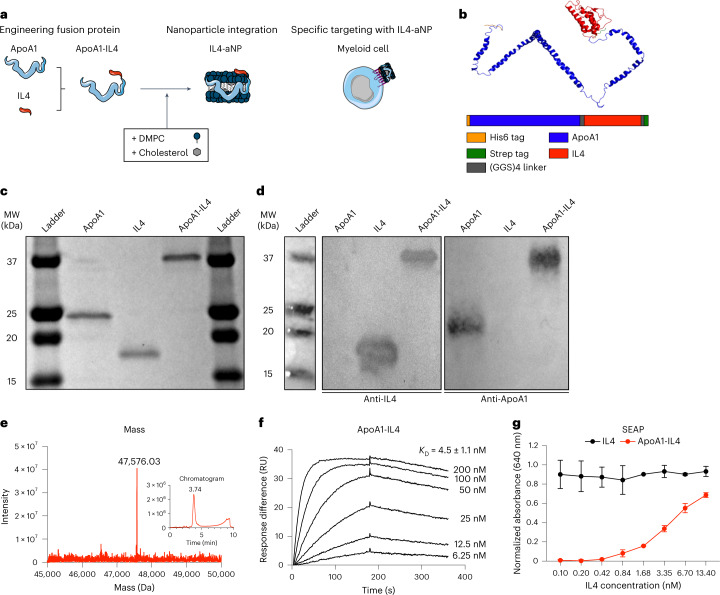
Engineering an apoA1–IL4 fusion protein. **a**, Schematic overview of apoA1-based fusion protein technology. **b**, Schematic of apoA1–IL4 fusion protein structure. **c**,**d** SDS–PAGE (**c**) and western blot (**d**) of recombinantly expressed proteins. Antibodies specific for endogenous IL4 and apoA1. **e**, Chromatogram and Q-ToF-MS spectrum of apoA1–IL4. **f**, Kinetics of apoA1–IL4 binding to IL4Rα using SPR. **g**, Activation of HEK-Blue cells expressing IL4Rα and IL13Rα1 by apoA1–IL4. Data are presented as mean ± s.d. DMPC, 1,2-dimyristoyl-*sn*-glycero-3-phosphocholine; GGS, glycine–glycine–serine; RU, resonance units.

Despite its ability to inhibit acute inflammation while simultaneously inducing trained immunity, the clinical translation of recombinant IL4 is hampered by its unfavourable pharmacokinetic properties. To overcome this limitation, we developed an apoA1-based fusion protein that readily integrates in lipid nanoparticles to yield IL4-containing nanoparticles (IL4-aNPs). ApoA1-based nanoparticles (aNPs) inherently accumulate in haematopoietic organs and efficiently target myeloid cells and their progenitors^16,23^ (Fig. 3a). Specifically, we designed a fusion protein consisting of human apoA1 and human IL4 (apoA1–IL4) connected via a flexible linker and flanked by two purification tags, a 6his tag located at the N-terminus and a strep tag at the C-terminus (Fig. 3b). We used molecular characterization techniques to confirm the nature and purity of apoA1–IL4. By performing sodium dodecyl sulphate–polyacrylamide gel electrophoresis (SDS–PAGE) on each purified protein sample, we confirmed the presence of protein with molecular weights of 25 kDa (apoA1), 18 kDa (IL4) and 37 kDa (apoA1–IL4) (Fig. 3c), while western blots indicated the presence of apoA1 and IL4 (Fig. 3d). Due to its amphiphilic nature, apoA1 and its derivatives run faster during gel electrophoresis compared to proteins of similar size. These observations were corroborated by quadrupole time-of-flight (Q-ToF) mass spectrometry (MS) showing a single mass peak at 47,576.03 Da corresponding to a molecular weight of 47,582.57 Da for apoA1–IL4 (Fig. 3e).

**Extended Data Fig. 3:**
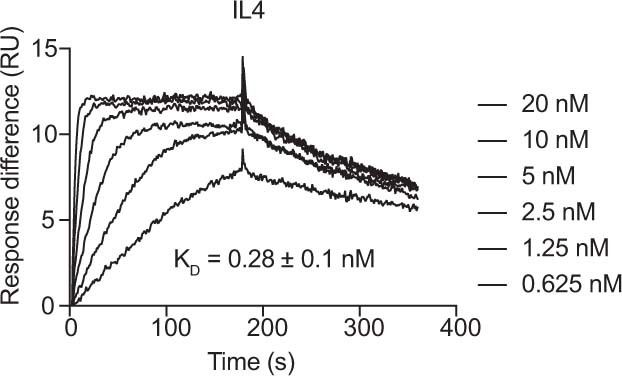
Engineering an apoA1–IL4 fusion protein. Kinetics of IL4 binding to IL4Rα using SPR.

Before integrating apoA1–IL4 in lipid nanoparticles, biophysical and cellular analyses—using surface plasmon resonance (SPR) and HEK-Blue IL4/IL13 (HEK-IL4) reporter cells—were performed to determine the preservation of biological activity after the extraction, purification and refolding process. We determined the equilibrium dissociation constants *K*_D_ of apoA1–IL4 against the human IL4 receptor alpha (IL4Rα) using SPR to be 4.5 ± 1.1 nM (Fig. 3f). HEK-IL4 cells possess an IL4Rα/STAT6-inducible reporter gene coding for secreted alkaline phosphatase (SEAP), which they prominently produced upon introduction of apoA1–IL4 to their culture wells. This indicates biological activity of apoA1–IL4 (Fig. 3g). Although fusion with apoA1 substantially altered the biophysical properties of IL4, enabling integration into lipid nanoparticles, binding to its receptor was preserved with a *K*_D_ of 0.28 ± 0.1 nM (Extended Data Fig. 3a). To summarize, we have developed an apoA1–IL4 fusion protein that preserves the biological activities of IL4 after extraction, purification and refolding and contains the desirable physicochemical features for its integration into lipid nanoparticles through apoA1.

### Integrating apoA1–IL4 in lipid nanoparticles

**Fig. 4:**
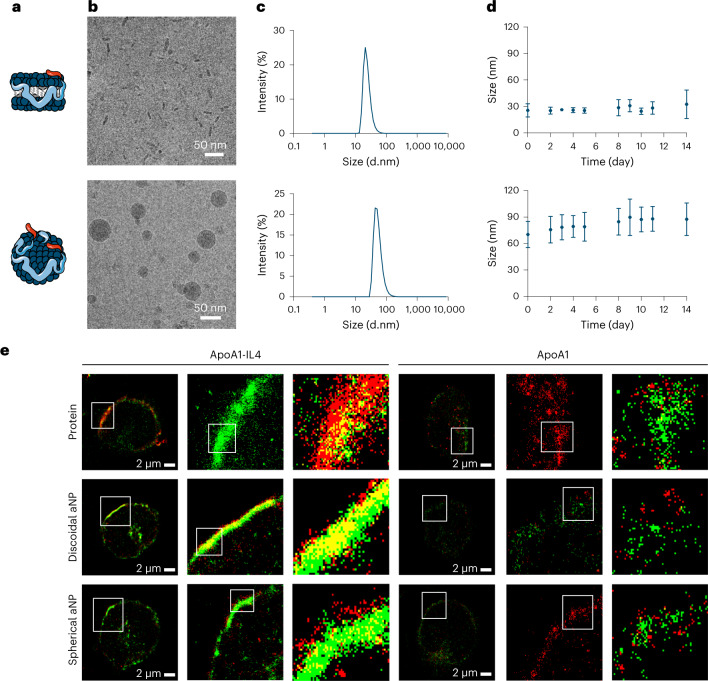
Integrating apoA1–IL4 in nanoparticles. **a**,**b**, Schematic representation (**a**) and cryo-TEM images (**b**) of discoidal (upper panel) and spherical IL4-aNPs (lower panel). **c**,**d**, IL4-aNP size distribution (**c**) and stability (**d**) of IL4-aNPs over time as determined by DLS. IL4-aNP size is reported as the number mean. **e**, Super-resolution fluorescence microscopy (dSTORM) images of human monocytes incubated with either fluorescently labelled apoA1(-IL4) or (IL4-)aNPs (red) and stained with anti-IL4Rα antibody (green). The co-localization between the proteins and IL4Rα can be appreciated in yellow. White ROIs are magnified in subsequent images on the right. Data are presented as mean ± s.d.

**Extended Data Fig. 4:**
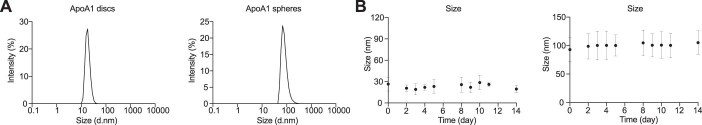
Integrating in aNP technology. (**a**) DLS evaluation of conventional aNPs particle size. (**b**) DLS stability of conventional aNPs over time.

To improve the pharmacokinetic properties of IL4 and its bioavailability to myeloid cells, we integrated the apoA1–IL4 fusion protein in lipid nanoparticles to yield IL4-aNPs^16^. Nanoparticles of different sizes and morphologies were obtained by varying the formulations’ compositions (Fig. 4a). The formation of discoidal and spherical nanoparticles was confirmed by cryogenic transmission electron microscopy (cryo-TEM) (Fig. 4b). In addition, we analysed nanoparticle size and stability in PBS for 14 days using dynamic light scattering (DLS) (Fig. 4c,d). IL4-aNPs remain stable for 14 days and have a similar size and stability compared to our previously reported conventional aNPs (Extended Data Fig. 4a,b).

Next, we investigated the interaction of IL4-aNPs with primary human monocytes. Direct stochastic optical reconstruction microscopy (dSTORM) analysis revealed the expression of IL4Rα (green) on the membrane. Furthermore, binding of bare apoA1, bare apoA1–IL4, aNPs and IL4-aNPs (red) were confirmed by the rim covering the cell surface. When focusing on a zoomed-in section of the membrane, we found that bare apoA1–IL4 and IL4-aNPs were associated with the IL4Rα, forming enriched co-clusters on the cell surface, which we did not observe for bare apoA1 and conventional aNPs (Fig. 4e). Together, DLS size stability assays, cryo-TEM and dSTORM analyses revealed that the integration of the apoA1–IL4 fusion protein in lipid nanoparticles yields biologically functional IL4-aNPs.

### Studying IL4, apoA1–IL4 and IL4-aNP formulations in mice

**Fig. 5:**
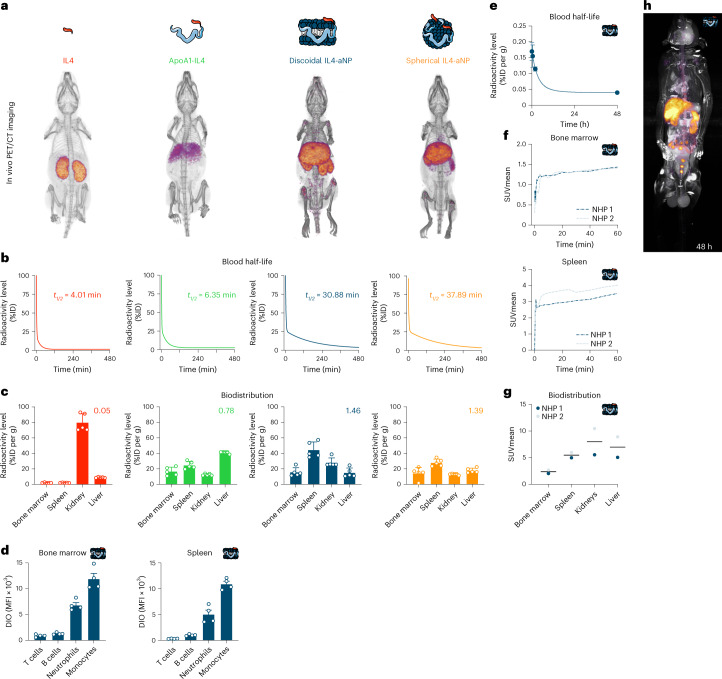
In vivo pharmacokinetics, biodistribution and safety profile after intravenous injection. **a**, PET-CT render at 24 h after injecting ^89^Zr-labelled constructs. **b**, ^89^Zr-labelled construct blood half-life (*n* = 5, as fitted with a two-phase decay function). ID, injected dose. **c**, Ex vivo gamma counting of tissues 24 h after ^89^Zr-labelled construct injection (*n* = 5), number represents ratio target to clearance organs. **d**, Cell type-specific biodistribution of DiO-labelled discoid IL4-aNPs in spleen and bone marrow, as measured by flow cytometry. **e**, ^89^Zr-IL4-aNP blood half-life in non-human primates. **f**, Organ SUV mean over time in ^89^Zr-IL4-aNPs-injected non-human primates (*n* = 2). **g**, Organ-specific SUV mean 48 h after ^89^Zr-IL4-aNPs injection in non-human primates (*n* = 2). **h**, PET-MRI scan of non-human primate 48 h after ^89^Zr-IL4-aNPs injection. Data are presented as mean ± s.d. where appropriate. DIO, 3,3′-dioctadecyloxacarbocyanine perchlorate; MFI, mean fluorescence intensity; NHP, non-human primate.

**Extended Data Fig. 5:**
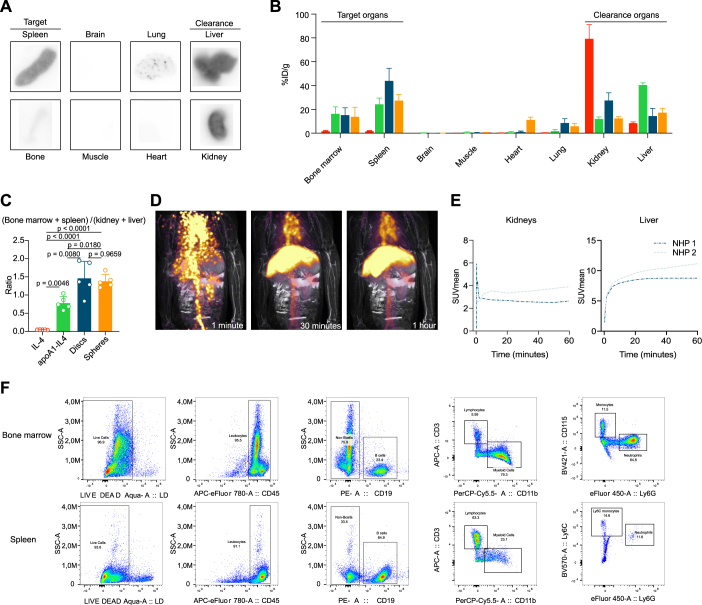
*In vivo* pharmacokinetics, biodistribution and safety profile. (**a**) Organ autoradiography 24 h after ^89^Zr-IL4-aNPs injection in mice. (**b**) Gamma count in vital organs 24 h after ^89^Zr-IL4-aNPs injection in mice (n = 5). (**c**) Uptake ratios by target organs over clearance organs in mice 2-way ANOVA with Turkey post-hoc analysis. (**d**) Dynamic PET/MRI scans of non-human primates at 1, 30, and 60 minutes after ^89^Zr-IL4-aNPs injection. (**e**) Organ-specific SUVmean in non-human primates over 1 hour following ^89^Zr-IL4-aNPs administration. (**f**) Gating strategy for bone marrow (top) and spleen (bottom) in experiments to measure cell type-specificity of DiO-labelled discoidal IL4-aNPs. Data are presented as mean ± SD where appropriate.

To investigate pharmacokinetics and biodistribution in C57BL/6 mice, we radiolabelled the protein component of four different IL4 therapeutics, namely, bare IL4, the bare apoA1–IL4 fusion protein and discoidal and spherical IL4-aNPs, with zirconium-89 (^89^Zr). Note that these experiments were performed with the human variant of IL4 that does not show biologic activity in mice. PET with computed tomography (PET-CT) imaging at 24 h post intravenous administration showed that ^89^Zr-IL4 and ^89^Zr-apoA1–IL4 accumulated mostly in the kidney and liver. In contrast, besides accumulating in the liver and kidney, ^89^Zr-IL4-aNPs accumulated in relatively higher amounts in immune-cell-rich organs, including the spleen and bone marrow (Fig. 5a). We performed ex vivo gamma counting to determine the nanomaterials’ blood half-lives and uptake in major organs (Fig. 5b,c), which we corroborated by autoradiography (Extended Data Fig. 5a). Comparison of uptake ratios by target organs (bone marrow + spleen) divided by clearance organs (kidney + liver) showed a significant increase in uptake ratio for IL4-aNP formulations compared to unformulated fusion protein and bare IL4 (Extended Data Fig. 5c). Next, we used flow cytometry to measure cell-type-specific biodistribution in target organs. 3,3′-Dioctadecyloxacarbocyanine perchlorate (DiO)-labelled discoidal IL4-aNPs accumulate in myeloid cells, most notably monocytes and neutrophils, in both spleen and bone marrow, while they do not (or only marginally) interact with lymphocytes (Fig. 5d). On the basis of its favourable (and myeloid-specific) uptake in haematopoietic organs, we selected the discoidal IL4-aNP formulation for further studies in non-human primates and translational models of inflammation and sepsis.

### IL4-aNP immunotherapy shows favourable uptake profile in non-human primates

To evaluate the clinical translatability of IL4-aNP immunotherapeutics, we determined their biodistribution and safety profile in non-human primates. Two non-human primates were injected intravenously with ^89^Zr-IL4-aNPs. Their in vivo behaviour was studied using fully integrated three-dimensional PET combined with magnetic resonance imaging (PET-MRI). After injection, dynamic PET-MRI (Extended Data Fig. 5d) showed rapid accumulation of IL4-aNPs in the liver, kidney (Extended Data Fig. 5e), spleen and bone marrow (Fig. 5e–h). In accordance with the mouse data, no undesirable uptake of IL4-aNPs was observed in non-target organs including the brain and heart (Extended Data Fig. 5b). Together, these results show that the favourable biodistribution and safety profile of IL4-aNP is retained across species, corroborating this immunotherapy’s translational potential.

### IL4-aNP therapy resolves immunoparalysis in vitro and in vivo

**Fig. 6:**
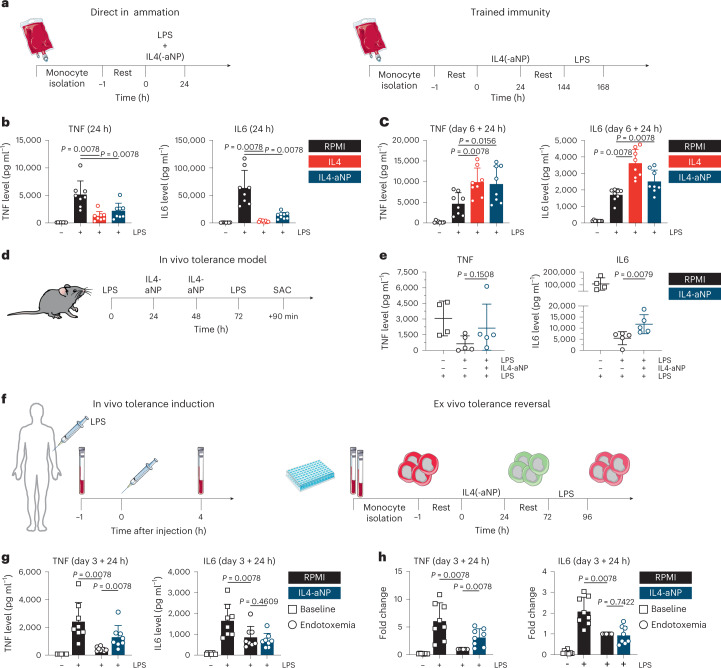
Immunological in vitro, in vivo and ex vivo therapeutic evaluation of IL4-aNPs. **a**, Schematic overview of the direct inflammation and trained immunity experiments in vitro. **b**. TNF and IL6 levels after 24 h stimulation of monocytes in the presence of IL4-aNPs. **c**, TNF and IL6 levels after re-stimulation of IL4(-aNP)-trained cells. **d**, Schematic overview of murine in vivo tolerance model, including IL4-nanotherapy. **e**, Serum TNF and IL6 levels following LPS re-challenge of mice treated with IL4_m_-aNPs. The Mann–Whitney *U* test was used for statistical comparisons. **f**, Schematic overview of human experimental endotoxaemia model, including ex vivo tolerance reversal. **g**, TNF and IL6 levels after ex vivo re-stimulation of human in vivo LPS-tolerized cells. **h**, TNF and IL6 fold increase after ex vivo re-stimulation of human in vivo LPS-tolerized cells. Data are presented as mean ± s.d.

**Extended Data Fig. 6:**
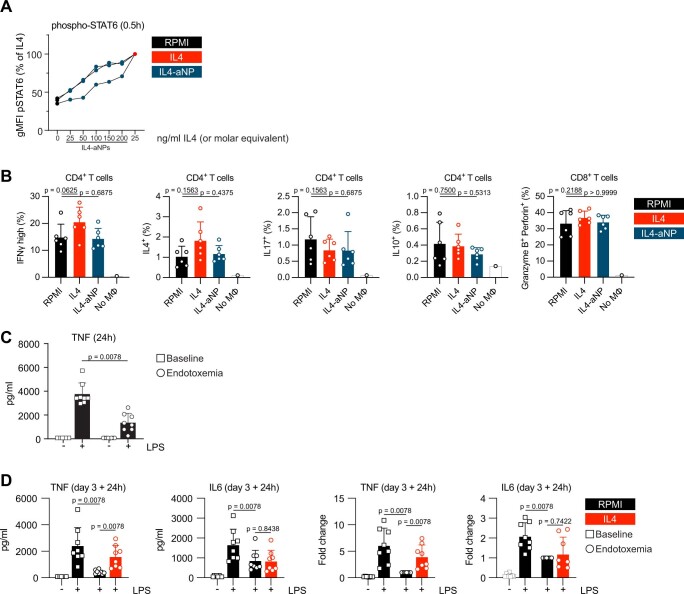
Therapeutic evaluation of IL4-aNPs. (**a**) Phosphorylation of STAT6, measured by flow cytometry after 30 minutes stimulation with RPMI, IL4, or different concentrations of IL4-aNPs. The data are expressed relative to the signal elicited by bare IL4. (**d**) T cell polarization assay by flow cytometry, after 7 days of mixed leukocyte reaction with IL4(-aNP)-trained macrophages or control. (**b**) TNF production following LPS-stimulation of monocytes isolated before and 4 hours after *in vivo* endotoxin challenge in humans. (**c**) TNF and IL6 levels after *ex vivo* re-stimulation of human *in vivo* LPS-tolerized cells (left) and the same data expressed as fold changes relative to the tolerant endotoxemia samples (right).

After establishing that natural IL4 simultaneously dampens acute inflammatory responses and induces a program of trained immunity, we assessed the effects of IL4-aNPs effects on monocytes in vitro (Fig. 6a). We based the dose for in vitro experiments on efficiency of phospho-STAT6 induction in primary human monocytes, relative to bare IL4 (Extended Data Fig. 6a). Indeed, IL4-aNPs (molar equivalent of 200 ng ml^−1^ bare IL4) significantly reduced TNF and IL6 production of LPS-stimulated monocytes (Fig. 6b), while enhancing long-term responsiveness of monocytes on day 6 (Fig. 6c). These data indicate that IL4-aNPs, similarly to IL4, suppress acute inflammation and induce trained immunity in vitro. Although in vivo biodistribution data show that IL4-aNPs specifically target myeloid cells (Fig. 5d), IL4-induced trained immunity changes surface marker expression of macrophages, which are antigen-presenting cells (Extended Data Fig. 1d,e). This in turn might affect polarization signals during T cell activation. To investigate these potential indirect effects of IL4-aNPs on T cells, allogenic naive T cells were cultured in the presence of IL4-trained macrophages. In this model, human leukocyte antigen mismatch causes antigen-unspecific T cell activation and polarization. No significant differences in the abundance of T cell subtypes, Th1 (CD4^+^ IFNγ^high^), Th2 (CD4^+^ IL4^+^), T_reg_ cells (CD4^+^ IL10^+^), Th17 (CD4^+^ IL17^+^) and cytotoxic T cells (CD8^+^ Granzyme B^+^ Perforin^+^) were observed between trained macrophages and controls (Extended Data Fig. 6d). Together, these findings indicate that IL4 training does not show the ability to skew T cells responses indirectly, suggesting a predominantly myeloid-specific effect.

Patients with sepsis may experience both hyper-inflammatory responses and immunoparalysis, creating a therapeutic paradox. Induction of trained immunity can theoretically be used to reverse immune tolerance, but this has not been translated to in vivo models^11^. One reason is that human IL4 does not show biologic activity in mice. We therefore designed and produced a chimeric fusion protein, consisting of human apoA1 and murine IL4 for formulation with lipids to yield IL4_m_-aNPs. Here we investigated whether IL4_m_-aNPs can reverse LPS-induced tolerance in mice. To that end, we intraperitoneally injected C57B/6 mice with LPS (0.1 mg kg^−1^) to induce immunoparalysis or PBS (as a control). We intravenously administered IL4_m_-aNPs (200 µg per dose) at 24 h and 48 h after LPS treatment. We re-challenged the mice with another intraperitoneal injection of LPS (0.1 mg kg^−1^) 72 h after the first challenge (Fig. 6d). Despite the constraints in effect size, treatment with IL4_m_-aNPs improved innate immune responses as signified by statistically significantly (*P* = 0.0079) increased serum IL6 concentrations following LPS re-challenge of tolerized mice (Fig. 6e). While TNF concentrations in some mice were also clearly elevated, statistical significance was not achieved (*P* = 0.1508) due to heterogeneity in the therapeutic response (Fig. 6e). Collectively, our in vitro and in vivo data show that IL4-aNP immunotherapeutics can reduce tolerance.

### Human endotoxaemia model

After we observed tolerance reversal in the mouse model, we substantiated these results with a model that more closely mimics human clinical immunoparalysis. We obtained blood from healthy individuals undergoing experimental human endotoxaemia, a standardized controlled model of systemic inflammation capturing hallmarks of both hyper-inflammatory and immunoparalytic phenotypes of sepsis (Fig. 6f). In this controlled human model, LPS is intravenously administered to healthy volunteers, leading to a systemic inflammatory response which is followed by tolerization of circulating monocytes, a phenomenon also observed in sepsis-induced immunoparalysis. Blood was collected before and 4 h after start of LPS administration. Monocytes isolated after LPS administration showed deficient cytokine production upon immediate re-exposure to LPS, indicating tolerization (Extended Data Fig. 6b). When the tolerant monocytes from the LPS-challenged volunteers were exposed ex vivo to either IL4 or IL4-aNPs for 24 h, they showed significantly improved production of TNF, but not IL6, upon re-stimulation with LPS on day 3 (Fig. 6g (concentration) and 6h (fold change) and Extended Data Fig. 6c). In contrast, untreated monocytes remained completely tolerant. Together, these data highlight the ability of IL4 and IL4-aNPs to at least partially reverse LPS tolerance ex vivo.

## Discussion

IL4 is generally considered to be an anti-inflammatory cytokine, yet its long-term effects on monocyte and macrophage function are unclear^15,24–26^. We initially expected that IL4 would inhibit trained immunity, similarly to IL37 and IL38 (refs. ^27,28^. In addition to its known inhibitory effects on acute inflammation, we observed that IL4 induces trained immunity as assessed by increased cytokine production responsiveness. Whereas the induction of trained immunity by IL4 was unexpected, our observations are in line with the activation of the mTOR signalling cascade by IL4, a central mechanism in trained immunity^10,29^. We subsequently investigated the long-term effect of IL4 pre-exposure as well as the capacity of IL4 to reverse immune tolerance induced by experimental endotoxaemia. In this context, the results show that an anti-inflammatory STAT6-dependent cellular program dominates during the acute exposure of cells to IL4 but that this shifts over time towards an mTOR-driven program of long-term trained immunity. IL4 training shows characteristics typically attributed to trained immunity, including enhanced cytokine production, epigenetic rewiring, increased metabolic activity and altered transcriptomic responses upon re-stimulation. These observations are in line with the growing evidence for a dynamic- and timing-dependent model of monocyte differentiation, such as that proposed in ref. ^30^.

The ability of IL4 to simultaneously suppress acute inflammation while inducing a trained immunity program, which has been reported to improve host defence^10^, can be potentially used to treat severe infections. For example, both sepsis and coronavirus disease 2019 (ref. ^31^) are characterized by a dysregulated immune response, creating a therapeutic paradox that requires both managing hyper-inflammatory responses and improving host-defence responses against (opportunistic) secondary infections. To take advantage of these features of IL4, we developed a nanoparticle protein-engineering strategy, thereby overcoming this cytokine’s unfavourable in vivo pharmacokinetic properties. Therefore, we trade the reduced IL4 potency of the fusion protein, a characteristic that can be improved in future studies by optimizing the protein and expression system as we did for the chimeric fusion protein, for the benefit of the substantially improved bioavailability and pharmacokinetics. We show that the aNP strategy favourably alters the blood half-life and biodistribution profile of IL4, resulting in an elevated neutrophil-specific and monocyte-specific accumulation in myeloid cell-rich organs such as the bone marrow and spleen, as earlier observed in models of trained immunity^32^. Furthermore, these studies have shown that aNPs consisting of 1,2-dimyristoyl-*sn*-glycero-3-phosphocholine (DMPC), cholesterol and apoA1 do not induce trained immunity, emphasizing that our findings are strictly related to the biological effect of IL4 (ref. ^33^). While we studied the biodistribution of aNPs containing human apoA1-IL4, we additionally developed the murine variant IL4_m_-aNP to evaluate in vivo efficacy. Indeed, we show that IL4_m_-aNPs can partially revert immunoparalysis in an LPS-induced sepsis mouse model. Although the data show restored innate immune responses, with a substantial increase of IL6 production and a clear trend towards increased TNF concentrations in the serum of mice treated with IL4_m_-aNP, full-blown dose-range-finding studies are required to expand the potential of IL4-aNP therapy in a range of immune-mediated diseases that are characterized by concurrent hyper-inflammation and immunoparalysis. It is encouraging that, using the human fusion protein, we showed that IL4-aNPs can reverse the immune tolerance of cells acquired from a human model mimicking clinical immunoparalysis.

We anticipate that the cytokine-nanoparticle technology may find uses in other myeloid-directed applications. The state of immunoparalysis is not unique to sepsis. Immuno-oncological applications might be considered, because cancer is also characterized by local pro-tumour inflammation and the simultaneous suppression of anti-tumour responses (often mediated by myeloid cells)^34^. Myocardial infarction and stroke are also characterized by sterile inflammation followed by immunoparalysis^35,36^, and patients with severe trauma suffer from a similar immune-paralytic condition^37^. In all these situations, reducing inflammation and overcoming immunoparalysis may be beneficial for patient recovery and preventing secondary infections. IL4-aNP technology might be developed into a therapeutic modality for the treatment of all these conditions.

## Methods

### Peripheral blood mononuclear cell and monocyte isolation

Buffy coats (Sanquin) or EDTA whole blood from healthy volunteers was acquired after obtaining written informed consent. The material was diluted at least 1:1 with calcium- and magnesium-free PBS (Lonza) and layered on top of Ficoll-Paque (GE Healthcare). Density-gradient centrifugation for 30 min at 615 *g* was used to separate the peripheral blood mononuclear cell (PBMC) interphase. Following three to five washes with cold PBS, PBMC yield and composition were assessed using a haemoanalyser (XN-450; Sysmex).

Negatively selected monocytes were obtained using MACS, according to the manufacturer’s instructions (MACS Pan monocyte isolation kit, human; Miltenyi Biotec). Monocyte yield and purity was assessed on a Sysmex haemoanalyser.

For some experiments (indicated in the text), monocytes were alternatively enriched from PBMCs by hyper-osmotic density-gradient centrifugation over Percoll (Sigma-Aldrich). About 150–200 × 10^6^ PBMCs were layered on top of a hyper-osmotic Percoll solution (48.5% *v*/*v* Percoll, 0.16 M NaCl, in sterile water) and centrifuged for 15 min at 580 *g*, room temperature (RT). The interphase was collected, washed once with cold PBS and re-suspended in RPMI.

### Primary human monocyte culture

All primary human monocytes and macrophages were cultured in RPMI-1640 with Dutch modifications (Invitrogen) which was further supplemented with GlutaMAX (2 mM; GIBCO), sodium pyruvate (1 mM; GIBCO) and gentamicin (50 μg ml^−1^; Centrafarm). This medium is further referred to as RPMI+++. In addition, 10% (*v*/*v*) human pooled serum was added to the medium during cell culture (also referred to as ‘cell culture medium’).

### In vitro model of trained immunity in primary human monocytes

To induce trained immunity in primary human monocytes, a previously optimized and published method was used^38,39^. Briefly, monocytes were adhered to a flat-bottom cell culture plate for 1 h and washed with warm PBS to remove any non-adherent cells and cell debris. Then, they were stimulated (‘trained’) for 24 h with one of the stimuli detailed in Supplementary Table 1, or medium only (‘untrained’ control).

For pharmacological inhibition experiments, the monocytes were pre-incubated for 1 h with one of the inhibitors described in Supplementary Table 2 before addition of the training stimulus.

Following the initial 24 h stimulation, the cells were washed with warm PBS, and warm cell culture medium was added. The monocytes were then allowed to rest and differentiate into macrophages for 5 days. On day 6, the induction of trained immunity was assessed. To this end, the cells were typically re-stimulated with LPS for an additional 24 h to elicit cytokine production. The supernatants were collected and stored at −20 °C until further analysis.

For most other trained immunity readout methods, the cells were collected as follows: first, the cells were incubated in Versene cell dissociation reagent (Life Technologies) for 30 min in a cell culture incubator. A cell scraper was then used to remove the cells from the culture plates. To maximize the yield, the culture plates were scraped a second time after adding ice-cold PBS. The macrophages were centrifuged for 10 min at 300 *g*, 4 °C and counted before continuing to downstream applications.

### Primary moDC generation

For experiments in which moDCs were compared to macrophages (untrained control or IL4-trained), moDCs were differentiated as follows. First, negatively selected monocytes were obtained as described above. Following 1 h adherence and a PBS wash, they were cultured in RPMI+++ with 10% HPS, further supplemented with IL4 (25 ng ml^−1^) and GM-CSF (1,000 IU ml^−1^; premium grade, Miltenyi Biotec). The cells were differentiated until day 6, with one medium refresh on day 3. On day 6, the non-adherent cells were collected in addition to the adherent cells (as described above). The moDCs and macrophages were then subjected to analysis by flow cytometry as described below.

### In vivo experimental human endotoxaemia model and ex vivo analyses

Eight healthy (as confirmed by medical history, physical examination and routine laboratory testing) male volunteers with ages ranging from 18 years to 35 years provided written informed consent to participate in experimental endotoxaemia experiments conducted at the research unit of the intensive care department of the Radboud University Medical Center. All study procedures were approved by the local ethics committee (CMO Arnhem-Nijmegen (Radboudumc), registration numbers NL71293.091.19 and 2019-5730) and were conducted in accordance with the latest version of the declaration of Helsinki.

A continuous endotoxin infusion regimen was used, as is described in detail elsewhere^40^. In short, participants were admitted to the research unit, and an antecubital vein and radial artery were cannulated to allow administration of fluids and endotoxin, and blood sampling and haemodynamic monitoring, respectively. A 3-lead ECG was recorded continuously throughout the experiment. After iso-osmolar pre-hydration (1.5 l NaCl 0.45% and glucose 2.5% administered intravenously in the hour before the start of endotoxin infusion), volunteers were intravenously challenged with a loading dose of 1 ng kg^−1^ bodyweight endotoxin (*Escherichia coli* lipopolysaccharide (LPS) type O113, lot no. 94332B1; List Biological Laboratories), directly followed by continuous infusion of 0.5 ng kg^−1^ h^−1^ for 3 h. Participants were monitored for 8 h after the endotoxin loading dose after which they were discharged from the research unit.

For this project, blood samples were obtained at two timepoints: 1 h before and 4 h after administration of the loading dose. Negatively selected monocytes were acquired as described above. The cells were adhered and stimulated for 24 h with recombinant human IL4, discoidal IL4-aNPs, LPS (to assess initial immune tolerance) or medium only (as a control). Following a PBS wash, the cells were rested in culture medium for 48 h and re-stimulated with LPS for an additional 24 h. Supernatants were collected and stored at −20 °C.

### Cytokine and lactate measurements

TNF, IL6 and IL1Ra were measured in cell culture supernatants using duoset ELISA kits (R&D Systems), according to the manufacturer’s instructions. For lactate measurements, a fluorometric assay was used. About 30 µl sample, medium control or known standard was added to a black 96-well plate. Then, 30 µl reaction mix (PBS pH 7.4, horse radish peroxidase (0.2 U ml^−1^), lactate oxidase (2 U ml^−1^), Amplex red (100 µM; Fisher scientific)) was added, and the reaction mixture was incubated for 20 min in the dark at RT. Immediately thereafter, fluorescence was measured at 530/25 nm and 590/35 nm. Gen5 software (v3.03, BioTek) was used in conjunction with Microsoft Excel to calculate cytokine and lactate concentrations in the original samples.

### Macrophage surface marker flow cytometry

Macrophages were collected as described above and transferred to a V-bottom 96-well plate for staining. The cells were centrifuged at 400 *g* for 5 min at 4 °C. The supernatant was removed, and the cells were washed once with 200 µl PBA (PBS pH 7.4, 1% *w*/*v* BSA (Sigma)).

Fc-receptors were blocked by incubation in PBS supplemented with 10% human pooled serum for 15 min at 4 °C. After washing once more, surface markers and viability were stained in a volume of 50 µl for 30 min at 4 °C, using the antibodies and viability dye described in Supplementary Table 3. Following two washes, the cells were re-suspended in 150 µl PBA and measured on a Cytoflex flow cytometer (Beckman Coulter) or BD FACSVerse system (BD Biosciences). Compensation was performed using VersaComp compensation beads (Beckman Coulter) for single antibody stains; a mixture of live and heat-killed cells was used for single stains of the viability dye (as per the manufacturer’s recommendations). Data analysis was performed in Flowjo (v10.7.1, BD Biosciences) Our gating strategy was as follows: first, a time gate was used if necessary. Then, single cell events were selected using subsequent FSC-A/SSC-A and FSC-A/FSC-H gates. Dead cells were removed from the analysis by selecting the viability dye-negative population. Geometric mean fluorescence intensities were calculated as a measure of surface marker expression.

### T cell polarization readout

For mixed lymphocyte reaction experiments, collected macrophages were used for subsequent T cell polarization assays. Allogeneic naive T cells were seeded with macrophages in a ratio of 10 T cells for every macrophage. The cells were cultured in flat-bottom 96-well plates for 7 days in standard cell culture medium. In this model, human leukocyte antigen mismatch causes non-specific activation of the T cell receptor. On the final day, the cells were stimulated with phorbol 12-myristate 13-acetate (25 ng ml^−1^) + ionomycin (0.5 µg ml^−1^) for 4 h in the presence of 100 ng ml^−1^ Brefeldin A, a ‘golgi plug’. The cells were collected and split over two flow-cytometry antibody panels (one for CD4 T cells and one for CD8; see also Supplementary Table 3). The cells were stained in a similar manner as described above, with an extra step for permeabilization of the T cells to allow for intracellular cytokine staining. This was performed using the fix and perm buffer set (eBioscience), according to the manufacturer’s instructions. The gating strategy was similar to what is described above, with the addition of a selection for CD3-positive events. The percentage of cells positive for hallmark cytokines of T cell polarization were calculated to estimate T cell subset proportions.

### Phospho-STAT6 measurement by flow cytometry

Monocytes were stimulated with RPMI, IL4 or different concentrations of IL4-aNPs (indicated in Extended Data Fig. 6a) for 20 min at 37 °C. The cells were transferred to a V-bottom 96-well plate and kept on ice for the duration of the staining procedure. After staining for viability and CD14 (in the manner described above), the cells were fixed and permeabilized using the fix and perm buffer set (eBioscience) for 45 min at 4 °C in the dark. The cells were washed twice with perm buffer and incubated overnight in freezer-chilled absolute methanol at −20 °C. Following two more washes in perm buffer the cells were stained for phospho-STAT6 using the antibody described in Supplementary Table 3, for 45 min at 4 °C in the dark. The cells were washed two more times in perm buffer and finally re-suspended in PBA for acquisition on the Cytoflex cytometer. The gating strategy was largely similar to the one for macrophage surface marker with the addition of a selection for CD14-positive events.

### Phagocytosis assay

Macrophages were collected as described above and incubated at 37 °C for 1 h with FITC-labelled *C. albicans* (kindly provided by M. Jaeger, Radboudumc) at an MOI of 1:5. The cells were washed two times with ice-cold PBA and kept on ice to halt the phagocytosis. The cells were stained for CD45 (Supplementary Table 3) for 30 min in the dark at 4 °C. Following two washes, trypan blue was added to a final concentration of 0.01% to quench extracellular FITC-*Candida*. The cells were then acquired on a Cytoflex flow cytometer.

During data analysis, CD45+ events were first selected to remove *Candida*-only events. Single cells were then gated on as described above, and the percentage of *Candida*-FITC positive macrophages in each sample was calculated.

### Seahorse metabolic analyses

Macrophages were collected as described above. The cells were re-suspended in RPMI+++ and seeded into overnight-calibrated cartridges at 10^5^ cells per well. After adhering for 1 h, the medium was changed to assay medium (Agilent; see below), and the cells were incubated for 1 h at 37 °C in ambient CO_2_ levels. Oxygen consumption rates and extracellular acidification rates were measured as proxies for glycolytic and mitochondrial metabolism, using a Seahorse XF Glycolysis Stress Test kit or a Seahorse XF Cell Mito Stress Test kit (both Agilent; measurements performed according to manufacturer’s instructions).

### RNA isolation, sequencing and analysis

Monocytes or macrophages were lysed in RLT buffer (Qiagen) and stored at −80 °C. RNA extractions were performed using RNeasy mini columns (Qiagen) with on-column DNAse I treatment (RNase-free; Qiagen). Preliminary quality control and measurements of concentration were performed using a Nanodrop apparatus. Samples were sent to the Beijing Genome Institute (BGI Denmark) for RNA sequencing using the DNBseq platform.

To infer gene expression levels, RNA sequencing reads were aligned to hg19 human transcriptome using Bowtie (v1.2)^41^. Quantification of gene expression levels as RPKM was performed using MMSEQ (v1.0.10)^42^. Reads and transcripts were normalized using DEseq2, and pair-wise comparisons were performed. Differentially expressed genes were identified using DEseq2 (v1.34.0) with fold change >2 and *P* < 0.05, with a mean RPKM >1 (ref. ^43^). To identify genes that were upregulated or attenuated by IL4 training, RPMI-d6 and IL4-d6 macrophages were compared with RPMI-d6+LPS and IL4-d6+LPS samples, respectively. Gene lists were merged and ranked on the basis of IL4-d6+LPS/RPMI-d6+LPS. Gene ontology and TF motif analysis was performed on gene promoters using the HOMER findMotifs tool (v4.11)^44^.

### ChIP

Macrophages were collected as described above and re-suspended in RPMI+++. The cells were fixed for 10 min in 1% methanol-free formaldehyde. The reaction was then quenched for 3 min by adding 125 mM glycine. The fixed cells were washed three times with ice-cold PBS, lysed at approximately 15 × 10^6^ cells per ml in lysis buffer (20 mM HEPES pH 7.6, 1% SDS, 1× protease inhibitor cocktail (Roche)), sonicated (Bioruptor Pico, Diagenode) and centrifuged (10 min, 16,060 *g*, at RT).

Aliquots of chromatin were de-crosslinked in 0.5× TBE buffer (supplemented with 0.5 mg ml^−1^ proteinase K (Qiagen)) for 1 h at 65 °C and run on a 1% agarose gel to confirm target fragment size of 200–800 bp.

The remaining chromatin was divided into ChIP and input samples. ChIP samples were diluted 10× in dilution buffer (16.7 mM Tris pH 8.0, 1.0% Triton, 1.2 mM EDTA, 167 mM NaCl, 1× protease inhibitor cocktail in Milli-Q), and 1 µg of ChIP-grade antibody (Diagenode) was added. The samples were rotated overnight at 4 °C.

Magnetic protein A/G beads (Dynabeads) were washed two times in dilution buffer supplemented with 0.15% SDS and 0.1% BSA. The washed beads were added to the ChIP samples and rotated at 4 °C for 1 h. The bead-bound chromatin was subsequently washed (rotation for 5 min, 4 °C) as follows: once with low-salt washing buffer (20 mM Tris pH 8.0, 1.0% Triton, 0.1% SDS, 2 mM EDTA, 150 mM NaCl in Milli-Q), two times with high-salt washing buffer (same as low-salt washing buffer but with 500 mM NaCl) and two times with no-salt washing buffer (20 mM Tris pH 8.0, 1 mM EDTA, in Milli-Q). Chromatin was eluted from the beads in elution buffer (0.1 M NaHCO_3_, 1% SDS, in Milli-Q) for 20 min, at RT. Input samples were diluted 12 times in elution buffer. After addition of NaCl (0.2 M) and proteinase K (0.1 mg ml^−1^), all samples were decrosslinked for at least 4 h on a shaking heatblock (65 °C, 1,000 r.p.m.). MinElute PCR purification columns (Qiagen) were used to purify DNA fragments. DNA fragments were stored at 4 °C until downstream analysis by qPCR.

### qPCR and analysis

qPCR analysis for ChIP samples and inputs was performed as follows. The SYBR green method was used to perform qPCR with the primers detailed in Supplementary Table 4. A comparative Ct method was used to compare ChIP against input samples and calculate relative abundance over a negative control region. *GAPDH* and the untranslated region of *ZNF* were respectively used as negative and positive controls for H3K9me3. *TNF* was interrogated using six primer pairs for AUC analysis as described previously^45^.

### Bacterial expression and protein purification

ClearColi BL21 (DE3) (Lucigen) were transformed with a pET20b(+)apoA1–IL4 expression vector. Transformed bacteria were inoculated in 40 ml lysogeny broth (Sigma-Aldrich) supplemented with 100 μg l^−1^ ampicillin and grown overnight at 37 °C. Subsequently, the overnight culture was inoculated in 2YT medium (16 g l^−1^ peptone, 10 g l^−1^ yeast extract and 10 g l^−1^ NaCl) supplemented with 100 µg l^−1^ ampicillin and grown at 37 °C. At the point that absorbance at 600 nm reached >1.5, 1.0 mM isopropyl β-d-thiogalacopyranoside was added to induce pET20b(+)apoA1–IL4 expression, and cells were incubated overnight at 20 °C. Cells were collected by centrifugation before preparation of lysates and purification.

### Bacterial lysis and protein purification

ApoA1–IL4 fusion protein expressing ClearColi cells were collected by centrifugation at 10,880 *g* and 4 °C for 10 min. Collected cells were re-suspended in PBS and centrifuged at 3,500 *g* and 4 °C for 15 min. Cells were lysed using 20 ml BugBuster Protein Extraction Reagent (Merck) and 20 µl Benzonase nuclease (Merck) per litre culture on a shaker for 30 min at RT. Cell lysates were centrifuged at 39,000 *g* and 4 °C for 30 min. Insoluble pellets were washed with 10 ml BugBuster per litre and centrifuged at 39,000 *g* and 4 °C for 20 min. Pellet containing inclusion bodies was re-suspended in extraction buffer (6 M guanidine hydrochloride, 50 mM potassium phosphate and 1 mM reduced glutathione) and incubated on a shaker for 15 min at RT. The suspension was centrifuged at 39,000 *g* and 4 °C for 30 min to remove insoluble fraction. Filtered soluble fraction was loaded on a nickel column and washed with 15 column volumes IMAC wash buffer. ApoA1–IL4 was unfolded and refolded on the nickel column using a linear gradient unfolding buffer 60 ml (7 M urea, 1 mM reduced glutathione, 0.1 mM oxidized glutathione, 50 mM potassium phosphate and 100 mM NaCl pH 6.8) to refolding buffer 60 ml (1 mM reduced glutathione, 0.1 mM oxidized glutathione, 50 mM potassium phosphate and 100 mM NaCl pH 6.8) at 2.5 ml min^−1^. Refolded apoA1–IL4 was eluted from the column with 0.5 M imidazole, 20 mM Tris and 0.5 M NaCl at pH 7.9. Eluate was collected, concentrated and further purified and buffer-exchanged via size exclusion chromatography (HiLoad 16/600 Superdex 75 Increase; GE Healthcare) equilibrated with PBS storage buffer. Fractions were analysed by SDS–PAGE, pooled, concentrated and snap-frozen in liquid nitrogen before storing at −80 °C. ApoA1–IL4 mass was confirmed by Q-ToF LC-MS (WatersMassLynx v4.1), using MagTran V1.03 for MS.

### Mammalian expression and purification of apoA1–IL4_m_

HEK293T cells were co transfected with fuGENE (Promega) including transfer vector pHR-apoA1–IL4_m_, packaging pCMVR8.74 and envelop pMD2.G in Opti-MEM (GIBCO) at 37 °C for 24 h. Cells were washed with DMEM supplemented with 2% heat inactivated FBS and incubated for 48 h. To obtain the lentivirus containing pHR-apoA1–IL4_m_, supernatant was centrifuged at 875 *g* to remove cell debris filtered through 0.45 µm PES syringe filter and centrifuged at 50,000 *g* for 2 h at 4 °C. Pellet containing pHR-apoA1–IL4_m_ lentivirus was re-suspended in culture medium, snap frozen in liquid nitrogen and sorted at −80 °C. HEK293F cells were transduced with pHR-apoA1–IL4_m_ containing lentivirus in transfection medium (DMEM, 10% HI FBS, 1× polybrene (Sigma-Aldrich) for 24 h. Subsequently, cells were cultured in expression medium (50% EX-CELL 293 Serum-Free Medium for HEK293 Cells (Merck) and 50% FreeStyle 293 Expression Medium (Thermo Fisher Scientific), supplemented with Glutamax, 1% Pen-Strep and 1 µg ml^−1^ doxycycline (Merck) on a shaker at 150 r.p.m. for 3 days at 37 °C. Culture supernatant containing apoA1–IL4_m_ was centrifuged at 3,500 *g*, 4 °C for 15 min and filtered through 0.22 µm PES syringe filter to remove cell debris. Filtered soluble fraction was loaded on a StrepTactin XT 4flow 5 ml column (Cytiva) and washed with 5 column volumes W-buffer (150 mM NaCl, 100 mM Tris, 1 mM EDTA pH 8) with flow rate 1–2 ml min^−1^. ApoA1–IL4_m_ was eluted from the column with W-buffer supplemented with 50 mM biotin. Eluate was collected, concentrated and snap-frozen in liquid nitrogen before storing at −80 °C. ApoA1–IL4_m_ mass was confirmed by Q-ToF LC-MS (WatersMassLynx v4.1), using MagTran V1.03 for MS.

### SDS–PAGE and western blot

To confirm fusion of apoA1 and IL4, 100 ng IL4 (BioLegend), apoA1 and apoA1–IL4 were loaded on a 4–20% polyacrylamide gel (Bio-Rad). After gel electrophoresis, samples were transferred to nitrocellulose membranes with blot buffer (10× TG buffer, 20% methanol). Subsequently, membranes were incubated with blocking buffer (5% milk, 0.1% tween in PBS (PBST)) overnight at 4 °C. The blots were incubated with primary monoclonal antibodies monoclonal anti-IL4 (HIL41, 1:200; sc-12723, Santa Cruz Biotechnology) and anti-apoA1 (B10, 1:100; sc-376818, Santa Cruz Biotechnology) for 1 h at 4 °C. After incubation with primary antibodies, membranes were washed and incubated with rabbit anti-mouse IgG (H+L)–HRP conjugate (1:5,000, 31457, Pierce). HRP-conjugated secondary antibodies were detected with TMB (Thermo Fisher Scientific) and visualized using the Image Quant gel imager (GE Healthcare).

### Surface plasmon resonance

SPR measurements were performed using a Biacore ×100 SPR system (GE Healthcare). Human IL4 receptor alpha-FC chimera (Biolegend) was immobilized on a protein G sensor chip (GE Healthcare). Log_2_ dilution concentration series consisted of apoA1–IL4 ranging from 200 nM to 6.25 nM and of human IL4 ranging from 20 nM to 0.65 nM. All samples were prepared in HPS-EP buffer (10 mM HEPES, 150 mM NaCl, 3 mM EDTA, 0.005% (*v*/*v*) P20 pH 7.4). Association was monitored for 180 s and dissociation for 180 s with a flow rate of 30 μl min^−1^. Sensor chip was regenerated with glycine 1.5 (10 mM glycine-HCl pH 1.5, GE Healthcare). Kinetics was determined by fitting the interaction SPR data for 1:1 binding.

### Human embryonic kidney 293 IL4 reporter cell assay

HEK-Blue IL4/IL13 cells were purchased from InvivoGen. This cell line has a fully active STAT6 pathway and carries a STAT6-inducible SEAP reporter gene. The HEK-Blue IL4/IL13 cells produce SEAP in response to IL4 and IL13. The levels of secreted SEAP can be determined with QUANTI-Blue (InvivoGen). About 180 μl DMEM with 10% FBS and 1% Pen-Strep containing 5 × 10^4^ cells was added per well in a 96-well-plate. Subsequently, 20 μl stimulus or vehicle was added, and cells were incubated for 20–24 h at 37 °C. Subsequently, 180 μl QUANTI-Blue was added per well to a separate 96-well-plate (flat-bottom), and 20 μl of the cell supernatant was added. The plate was incubated for 1–3 h at 37 °C, and absorbance at 640 nm was measured on a Tecan Spark plate reader to determine SEAP levels.

### Formulating nanoparticles

All phospholipids were purchased from Avanti Polar Lipids. Four different aNPs were formulated. For discoidal aNPs from stock solutions (10 mg ml^−1^) in chloroform, DMPC (133.5 μl) and cholesterol (Sigma-Aldrich) (7.5 μl), and for spherical aNPs, POPC (66.5 μl), PHPC (17.5 μl), cholesterol (4.5 μl) and tricaprylin (Sigma-Aldrich) (2.79 μl from 0.956 g ml^−1^ stock) were combined in a glass vial and dried under vacuum. The resulting film was re-dissolved in an acetonitrile and methanol mixture (95:5%, 800 μl total volume). For formulation based on apoA1, cholesterol (15 μl) was used. Separately, a solution of apoA1 protein in PBS (6 ml, 0.1 mg ml^−1^), apoA1–IL4 protein in PBS (6 ml, 0.17 mg ml^−1^) or apoA1–IL4_m_ in PBS (6 ml, 0.18 mg ml^−1^) was prepared.

Both solutions were simultaneously injected using a microfluidic pump fusion 100 (Chemyx) into a Zeonor herringbone mixer (Microfluidic Chipshop, product code 10000076) with a flow rate of 0.75 ml min^−1^ for the lipid solution and a rate of 6 ml min^−1^ for the apoA1 solution. The obtained solution was concentrated by centrifugal filtration using either a 10 kDa MWCO for discoidal and a 100 kDa MWCO for spherical aNPs Vivaspin tube at 3,500 *g* to obtain a volume of 1 ml. PBS (5 ml) was added, and the solution was concentrated to 5 ml; this was repeated twice. The washed solution was concentrated to approximately 1.5 ml and filtered through a 0.22 μm PES syringe filter to obtain the finished aNPs. Protein concentration in aNP samples was quantified with the Pierce BCA Protein Assay Kit (Thermo Fisher Scientific). To formulated fluorescent aNPs, 0.5 mg of DiOC18(3) dye (DiO) (Thermo Fisher Scientific) was dissolved in the chloroform solution used to prepare the lipid film.

### Determining aNP size and dispersity by DLS

Obtained aNP formulations in PBS were filtered through a 0.22 µm PES syringe filter and analysed by DLS on a Malvern Zetasizer Nano ZS analyser. Values are reported as the mean number average size distribution.

### Radiolabelling aNPs

IL4, apoA1–IL4 and IL4-aNPs were incubated with two molar excesses of DFO-p-NCS (5 mg ml^−1^ in DMSO) for 2 h washed three times using a 10 kDa MWCO Vivaspin tubes to remove any unreacted DFO-p-NCS. For radiolabelling, DFO coupled proteins and aNPs were incubated with ^89^Zr at 37 °C using a thermomixer at 600 r.p.m. for 1 h and washed three times using 10 kDa MWCO Vivaspin tubes to remove any unreacted ^89^Zr.

### Cryo-TEM of IL4-aNPs

First, the surface of 200-mesh lacey carbon supported copper grids (Electron Microscopy Sciences) was plasma treated for 40 s using a Cressington 208 carbon coater. Subsequently, 3 ml of IL4-aNPs sample (~1 mg protein per ml) was applied on a grid and vitrified into a thin film by plunge vitrification in liquid ethane by using an automated robot (FEI Vitrobot Mark IV). Cryo-TEM imaging was performed on the cryoTITAN (Thermo Fisher Scientific), equipped with a field emission gun, a post-column Gatan imaging filter (model 2002) and a post-GIF 2k × 2k Gatan CCD camera (model 794).The images were acquired at 300 kV acceleration voltage in bright-field TEM mode with zero-loss energy filtering at either 6,500× (dose rate of 1.64 electrons A^−2^ s^−1^) or 24,000× magnification (dose rate of 11.8 electrons A^−^^2^ s^−1^) and 1 s acquisition time.

### Super-resolution fluorescence microscopy of the interactions of IL4-aNPs with IL4 receptor in human monocytes

Human monocytes were isolated from a healthy donor’s peripheral blood as described above. About 100,000 monocytes were seeded per well on a cell-culture-treated chambered coverslip (µ-Slide 8-well, IBID). After 2 h of incubation at 37 °C (cell attachment), the cells were incubated for 2 h at 37 °C with Cy5-labelled variant of either bare apoA1 or apoA1–IL4, discoidal aNPs or IL4-aNPs, and spherical aNPs or IL4-aNPs. Subsequently, the cells were washed with PBS and fixed with 4% PFA for 20 min. IL4 receptor was stained with a polyclonal rabbit IgG1 anti-human IL4R (Thermo Fisher Scientific; 1:100 dilution) primary antibody for 24 h at 4 °C, followed by a goat anti-rabbit Alexa Fluor 488-conjugated secondary antibody (Thermo Fischer Scientific; dilution 1:500) for 1 h at room temperature. The stained cells were stored in PBS at 4 °C. For the dSTORM, the cells were immersed in GLOXY imaging buffer (40 μg ml^−1^ catalase, 0.5 mg ml^−1^ glucose oxidase, 5% glucose and 0.01 M cysteamine in PBS, pH 8.0) for a few minutes before and during the imaging. The acquisition was performed in total internal reflection fluorescence mode, using ONI Nanoimager (ONI). It is equipped with a 100×/1.4NA oil immersion objective and a sCMOS camera, and in this study, 488 nm (200 mW) and 640 nm (1000 mW) lasers were used. About 10,000 frames were acquired with a 10 ms exposure time with the field of view of 50 × 80 µm. Raw data were processed using the ThunderSTORM software (v1.3)^46,47^, yielding images with a spatial resolution of 10 nm.

### Animal models

Female C57BL/6 J mice (approximately 8–11 weeks old and approximately 20 g) were purchased from Charles River Germany. For non-human primate studies, two male cynomolgus monkeys (*Macaca fascicularis*) were used, aged 15 years and 16 years. All animals were co-housed in climate-controlled conditions, respectively, at 20–24 °C, 45–65% humidity with 12 h light–dark cycles and provided water ad libitum. Mice were fed a standard chow diet, and non-human primates were fed Teklad Global 20% Protein Primate Diet. Animal care and experimental procedures were based on approved institutional protocols from the Icahn School of Medicine at Mount Sinai. All mice were randomly assigned to experimental groups.

### Pharmacokinetics and biodistribution in mice and non-human primates

C57BL/6 mice were intravenously injected with ^89^Zr-labelled IL4 variants, respectively, IL4 (53.6 ± 6.6 μCi), apoA1–IL4 (30.1 ± 0.9 μCi), discoidal IL4-aNPs (146.1 ± 46.5 µCi) and spherical IL4-aNPs (108.6 ± 16.9 μCi). Two non-human primates were injected with discoidal ^89^Zr-labelled IL4-aNPs (1079 μCi and 682 μCi). At predetermined time points, 1, 2, 5, 10 and 30 min and 1, 2, 4, 8 and 24 h for mice and 5, 30 and 90 min and 48 h for non-human primates, after injection blood was drawn and weighed, and radioactivity was measured using a Wizard^2^ 2480 automatic gamma counter (Perkin Elmer). Data were corrected for radioactive decay, and percentage of injected dose per gram of blood (%ID per g) was calculated. Data were fitted using a nonlinear two-phase decay regression in GraphPad Prism, and weighted blood half-life was calculated via the equation (% fast × *t*_1/2_ fast + % slow × *t*_1/2_)/100. Biodistribution in mice was determined 24 h post injection. After PBS perfusion, tissues of interest were collected and weighed, and radioactivity was measured using a Wizard^2^ 2480 automatic gamma counter (Perkin Elmer). Data were corrected for radioactive decay, and percentage of injected dose per gram of tissue (%ID per g) was calculated.

### PET-CT imaging of aNP biodistribution in mice

C57BL/6 mice were injected intravenously with ^89^Zr-labelled IL4 variants, respectively, IL4 (53.6 ± 6.6 μCi), apoA1–IL4 (30.1 ± 0.9 μCi), discoidal IL4-aNPs (146.1 ± 46.5 μCi) and spherical IL4-aNPs (108.6 ± 16.9 μCi). After 24 h, mice were anaesthetized using 1.0% isoflurane in O_2_ at a flow rate of ∼1.0 l min^−1^. PET-CT scans were acquired using a Mediso nanoScan PET-CT (Mediso). A whole-body CT scan was executed (energy, 50 kVp; current, 180 μAs; isotropic voxel size, 0.25 mm) followed by a 20 min PET scan. Reconstruction was performed with attenuation correction using the TeraTomo 3D reconstruction algorithm from the Mediso Nucline software v3.04.020.0000. The coincidences were excluded by an energy window between 400 keV and 600 keV. The voxel size was isotropic with 0.4 mm width, and the reconstruction was applied for four full iterations, six subsets per iteration.

### Autoradiography

Tissues were placed in a film cassette against a phosphorimaging plate (BASMS-2325, Fujifilm) at −20 °C to determine the radioactivity distribution. The plates were read at a pixel resolution of 25 mm with a Typhoon 7000IP plate reader (GE Healthcare).

### Cellular specificity flow cytometry

For cellular specificity, mice were intravenously injected with DiO-labelled IL4-aNPs that was allowed to circulate for 24 h. Subsequently, mice were killed, and single cell suspensions were created from blood, spleen and bone marrow as previously described. Cell suspensions were incubated with anti-CD115, anti-CD11b, anti-Ly6C, anti-Ly6G, anti-CD19, anti-CD45, anti-CD11c, anti-CD3 and anti-F4/80. Live or dead Aqua was used as viability stain. Cells were subsequently washed and re-suspended in FACS-buffer. All data were acquired on an Aurora 5 l flow cytometer (Cytek Biosciences). DiO-IL4-aNPs were detected in the FITC channel.

### PET-MRI non-human primate biodistribution

After overnight fasting, non-human primates were anaesthetized using ketamine (5 mg kg^−1^) and dexmedetomidine (0.0075–0.015 mg kg^−1^). Non-human primates were injected with 1.114 mCi and 0.682 mCi discoidal ^89^Zr-labelled IL4-aNPs, at a dose of approximately 0.1 mg kg^−1^. Dynamic PET imaging as performed for 60 min following infusion, and additional static PET-MRI scans were performed at 1 h and 48 h after injection. Furthermore, blood was drawn during imaging at 5 min, 30 min and 120 min after injection. PET and MRI images were acquired using a 3T PET-MRI system (Biograph mMR, Siemens Healthineers). Beginning concurrently with the injection of aNPs, dynamic PET imaging was performed using one bed position covering the chest and abdomen. MR imaging parameters were as follows: acquisition plane, coronal; repetition time, 1,000 ms; echo time, 79 ms; number of slices, 144; number of averages, 4; spatial resolution, 0.5 × 0.5 × 1.0 mm^3^; and acquisition duration, 42 min and 42 s. After dynamic PET image acquisition, static whole-body PET images were acquired from the cranium to the pelvis, using 4 consecutive bed positions of 15 min each. Simultaneously with each bed, MR images were acquired as described above, except using only 1.4 signal averages, number of slices 160 and spatial resolution 0.6 × 0.6 × 1.0 mm^3^ (acquisition duration, 14 min 56 s per bed). Whole-body PET and MR imaging was also performed at 48 h after injection, using 4 PET bed positions of 30 min each, with MR parameters as follows: acquisition plane, coronal; repetition time, 1,000 ms; echo time, 79 ms; number of slices, 224; number of averages, 2; spatial resolution, 0.6 × 0.6 × 1.0 mm^3^; and acquisition duration, 29 min and 56 s. Whole-body MR images from each bed were automatically collated together by the scanner. After acquisition, PET raw data from each bed were reconstructed and collated together offline using the Siemens proprietary e7tools with an ordered subset expectation maximization algorithm with point spread function correction for 3 iterations and 24 subsets. Also, Gaussian filter of 4 mm was applied to the images. A three-compartment (soft tissue, lung and air) attenuation map was used for attenuation.

### Imaging-based analysis of the IL4-aNP biodistribution in non-human primates

Image analysis was performed using Osirix MD, version 11.0. Whole-body MR images were fused with PET images and analysed in a coronal plane. Regions of interest (ROIs) were drawn on tissues of interest including spleen, liver, kidneys, lungs, heart, cerebellum and cerebrum, which were traced in their entirety, and bone marrow uptake was determined using three vertebrae in the lumbar spine. For each ROI, mean standardized uptake values (SUVs) were calculated. Discoidal ^89^Zr-labelled IL4-aNP uptake per organ was expressed as the average of all mean SUV values per organ.

### In vivo tolerance model

For in vivo tolerance model, 11-week-old female C57BL/6 mice were intraperitoneal tolerized with 0.1 mg kg^−1^ body weight LPS. At 24 h and 48 h, mice were treated intravenously with either 200 µg IL4_m_-aNPs or PBS. Subsequently, mice were re-challenged with intraperitoneal 0.1 mg kg^−1^ LPS injection at 72 h. After 90 min, mice were killed, blood was collected for ELISA and single cell suspensions were created from blood, spleen and bone marrow as previously described. For staining protocol, blood samples for ELISA were allowed to clot at RT for 30 min. Serum was taken after centrifugation at 1,000 *g* for 10 min at 4 °C. Mouse TNF and IL6 ELISAs (Biolegend) were performed according to manufacturer’s protocols. Animal care and experimental procedures were based on approved institutional protocols from the Nijmegen Animal Experiments Committee.

### Statistical analysis

Data are shown as mean ± s.d., unless otherwise indicated. Individual data points in graphs are biological replicates, not technical repeats. The number of data points can be clearly discerned in each figure or *n* is indicated in the figure legend. Unless otherwise indicated, statistical analyses were performed in Graphpad Prism (v9, Graphpad Software). For trained immunity and acute stimulation experiments with primary human monocytes (paired, non-parametric), Wilcoxon signed-rank tests were used. Statistical methods for RNA-sequencing analysis are described above. Two-sided *P* values under 0.05 were considered statistically significant.

### Reporting summary

Further information on research design is available in the Nature Portfolio Reporting Summary linked to this article.

## Supplementary information

Supplementary Information Supplementary Tables 1–5, and unprocessed SDS–PAGE gels and western blots for Fig. 3.

Reporting Summary

## Data Availability

The main data supporting the results in this study are available within the paper and its Supplementary Information. The raw and analysed datasets generated during the study are available for research purposes from the corresponding authors on reasonable request. Raw RNA sequencing data are available from the NCBI Gene Expression Omnibus under accession number GSE185433.
