# Supplementary material for: Resolving sepsis-induced immunoparalysis via trained immunity by targeting interleukin-4 to myeloid cells
Source: Nat Biomed Eng. 2023 Jun 8;7(9):1097–112. doi: 10.1038/s41551-023-01050-0 (PMC10504080; doi:10.1038/s41551-023-01050-0)

nature biomedical engineering

Article https://doi.org/10.1038/s41551-023-01050-0

Resolving sepsis-induced immunoparalysis via trained immunity by targeting interleukin-4 to myeloid cells

In the format provided by the authors and unedited

nature biomedical engineering

Article https://doi.org/10.1038/s41551-023-01050-0

Resolving sepsis-induced immunoparalysis via trained immunity by targeting interleukin-4 to myeloid cells

In the format provided by the authors and unedited

Supplementary Table 1 I Stimuli for primary human monocytes and in vivo experiments.

Stimulus	Concentration in experiment	Source	#
Recombinant human IL4 (CHO-expressed)	25 ng/ml	R&D Systems	6507-IL
C. albicans-derived β-glucan	1 µg/ml	Kindly provided by David Williams (TN, USA)	-
GM-CSF (premium grade)	1000 IU/ml	Miltenyi biotec	130-093-864
E. coli LPS serotype O55:B5; further purified as described previously [47] to specifically activate TLR-4	10 ng/ml	Kindly prepared by Heidi Lemmers, Radboudumc	-
E. coli LPS serotype O55:B5 (for in vivo experiments)	0.1 mg/kg	Sigma	L2880-25MG
ApoA1-IL4 fusion protein	Molar equivalent of 200 ng/ml IL4	Prepared by D.P.S.	-
IL4-aNP (discoidal and spherical)	Molar equivalent of 200 ng/ml IL4	Prepared by D.P.S.	-
ApoA1-$IL4_m$ fusion protein	-	Prepared by D.P.S. and A.d.D.	
$IL4_m$-aNP (discoidal)	200 µg/mouse	Prepared by D.P.S. and A.d.D.	

Supplementary Table 2 I Inhibitors for primary human monocytes.

Inhibitor (target)	Concentration in experiment	Source	#
Wortmannin (PI3K)	100 nM	Invivogen	Tlrl-wtm
Torin-1 (mTOR)	5 μM	Invivogen	Inh-tor1
Cyproheptadine (SET7)	100 μM	Selleckchem	S2044
AS1517499 (STAT6)	300 nM	Sigma	SML1906

Supplementary Table 3. Flow cytometry antibodies for experiments with primary human monocytes/macrophages (and MLR experiments).

Target	Clone	Fluorophore	Source	Dilution	#	Assay
CD80	2D10	AF488	Biolegend	1:20	305214	Surface markers
CD14	M5E2	PE	Biolegend	1:20	301806	Surface markers
CD206	15-2	BV421	Biolegend	1:20	321126	Surface markers
CD200R	OX108	AF647	Biorad	1:10	MCA2282T	Surface markers
Viability	Fixable viability dye	eFluor780	Thermo Fisher Scientific	1:1000	65-0865	Surface markers
CD45	HI30	BV510	Biolegend	1:20	304036	Phagocytosis
Candida albicans	-	FITC	Kindly provided by Dr. Martin Jaeger, Radboudumc	MOI 1:5		Phagocytosis
CD1c	L161	BV421	Biolegend	1:33	331526	moDC comparison
Viability	Fixable viability dye	eFluor506	Thermo Fisher Scientific	1:1000	65-0866	moDC comparison
IFNγ	B27	BV421	BD	1:50	562988	MLR (T cell)
IL4	7A3-3	PE	Miltenyi	1:25	130-091-647	MLR (T cell)
IL10	JES3-9D7	VioB515	Miltenyi	1:25	130-108-100	MLR (T cell)

IL17	BL168	AF647	Biolegend	1:25	512310	MLR (T cell)
CD4	RPA T4	PerCP	Biolegend	1:25	300528	MLR (T cell)
CD8	RPA T8	FITC	BD	1:33	555366	MLR (T cell)
Granzyme B	QA16A02	PE	Biolegend	1:33	377208	MLR (T cell)
Perforin	dG9	AF647	Biolegend	1:33	308110	MLR (T cell)
pSTAT6	CHI2S4N	PE	eBioscience	1:10	12-9013-42	pSTAT6

Supplementary Table 4 I Primers for ChIP-qPCR analysis.

Target	Forward (5' – 3')	Reverse (5' – 3')
ZNF UTR	AAGCACTTTGACAACCGTGA	GGAGGAATTTTGTGGAGCAA
GAPDH	CCCCGGTTTCTATAAATGAGC	AAGAAGATGCGGCTGACTGT
TNF (1)	AGAGGACCAGCTAAGAGGGA	AGCTTGTCAGGGGATGTGG
TNF (2)	CAGGCAGGTTCTCTTCCTCT	GCTTTCAGTGCTCATGGTGT
TNF (3)	GTGCTTGTTCCTCAGCCTCT	ATCACTCCAAAGTGCAGCAG
TNF (4)	TGTCTGGCACACAGAAGACA	CCCTGAGGTGTCTGGTTTTC
TNF (5)	AGCCAGCTGTTCCTCCTTTA	TTAGAGAGAGGTCCCTGGGG
TNF (6)	TGATGGTAGGCAGAACTTGG	ACTAAGGCCTGTGCTGTTCC

Supplementary Table 5 I Flow cytometry antibodies for cell specificity biodistribution in mice.

Target	Clone	Fluorophore	Source	Dilution	#
CD115	AFS98	BV421	eBiosciences	1:200	17-1152-82
CD11b	M1/70	PerCP-Cy5.5	ThermoFisher	1:200	62-0112-82
Ly6C	AL-21	BV570	BioLegend	1:200	128017
Ly6G	1A8	eFluor 450	eBiosciences	1:200	48-9668-82
CD19	1D3	PE	BD PharMingen	1:200	557399
CD45	30-F11	APC-eFluor 780	BioLegend	1:200	103138
CD11c	N418	BB770	BioLegend	1:200	117328
CD3	17A2	APC	BioLegend	1:200	100249
F4/80	BM8	PE-Cy7	BioLegend	1:200	123110
Viability	Zombie Aqua Fixable Viability Kit	Zombie Aqua	BioLegend	1:1000	423101

Unprocessed SDS-PAGE gels and Western blots for Fig. 3

Support Figure 3C

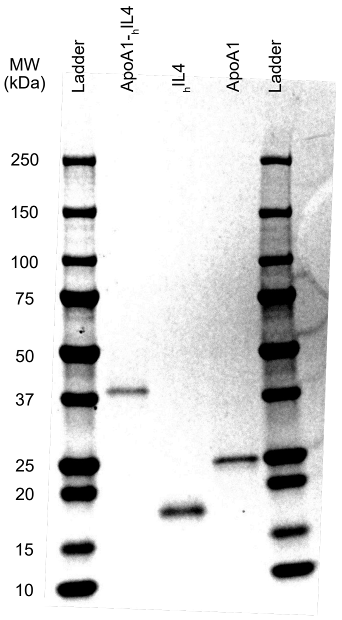

Support Figure 3D

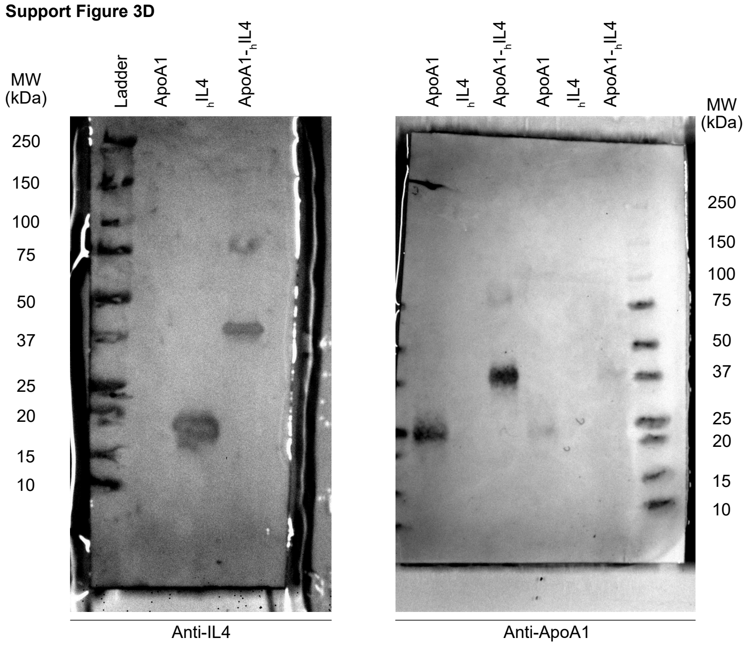

Supplement: Supplementary file 1 — Supplementary Tables 1–5, and unprocessed SDS–PAGE gels and western blots for Fig. 3. [file 41551_2023_1050_MOESM1_ESM.pdf]
