# Supplementary material for: Resolving sepsis-induced immunoparalysis via trained immunity by targeting interleukin-4 to myeloid cells
Source: Nat Biomed Eng. 2023 Jun 8;7(9):1097–112. doi: 10.1038/s41551-023-01050-0 (PMC10504080; doi:10.1038/s41551-023-01050-0)
Supplement: Supplementary file 2 — Reporting Summary [file 41551_2023_1050_MOESM2_ESM.pdf]

nature portfolio

Corresponding author(s): Mihai Netea and Willem Mulder

Last updated by author(s): May 1, 2023

Reporting Summary

Nature Portfolio wishes to improve the reproducibility of the work that we publish. This form provides structure for consistency and transparency in reporting. For further information on Nature Portfolio policies, see our Editorial Policies and the Editorial Policy Checklist.

Statistics

For all statistical analyses, confirm that the following items are present in the figure legend, table legend, main text, or Methods section.

n/a	Confirmed	
☐	☒	The exact sample size (n) for each experimental group/condition, given as a discrete number and unit of measurement
☐	☒	A statement on whether measurements were taken from distinct samples or whether the same sample was measured repeatedly
☐	☒	The statistical test(s) used AND whether they are one- or two-sided<br>*Only common tests should be described solely by name; describe more complex techniques in the Methods section.*
☒	☐	A description of all covariates tested
☐	☒	A description of any assumptions or corrections, such as tests of normality and adjustment for multiple comparisons
☐	☒	A full description of the statistical parameters including central tendency (e.g. means) or other basic estimates (e.g. regression coefficient) AND variation (e.g. standard deviation) or associated estimates of uncertainty (e.g. confidence intervals)
☐	☒	For null hypothesis testing, the test statistic (e.g. F, t, r) with confidence intervals, effect sizes, degrees of freedom and P value noted<br>*Give P values as exact values whenever suitable.*
☒	☐	For Bayesian analysis, information on the choice of priors and Markov chain Monte Carlo settings
☒	☐	For hierarchical and complex designs, identification of the appropriate level for tests and full reporting of outcomes
☒	☐	Estimates of effect sizes (e.g. Cohen's d, Pearson's r), indicating how they were calculated

Our web collection on statistics for biologists contains articles on many of the points above.

Software and code

Policy information about availability of computer code

Data collection	No software was used to collect data.
Data analysis	This is described in Methods. Briefly: unless otherwise indicated, statistical analyses were performed in Graphpad Prism (V9, Graphpad Software). To infer gene-expression levels, RNA-seq reads were aligned to hg19 human transcriptome using Bowtie. Quantification of gene-expression levels as RPKM was performed using MMSEQ. Reads/transcript were normalized using DEseq2 and pair-wise comparisons were performed. Gene ontology and TF motif analysis was performed on gene promoters using the HOMER findMotifs tool. Flow-cytometry data analysis was performed in Flowjo (v10.7.1, BD Biosciences). Cytokine and lactate concentrations in cell culture supernatants were calculated using a combination of Gen5 software (version 3.03, BioTek) and Microsoft Excel. STORM-images were analyzed using ThunderSTORM sortware (v1.3).

For manuscripts utilizing custom algorithms or software that are central to the research but not yet described in published literature, software must be made available to editors and reviewers. We strongly encourage code deposition in a community repository (e.g. GitHub). See the Nature Portfolio guidelines for submitting code & software for further information.

Data

Policy information about availability of data

All manuscripts must include a data availability statement. This statement should provide the following information, where applicable:

- Accession codes, unique identifiers, or web links for publicly available datasets
- A description of any restrictions on data availability
- For clinical datasets or third party data, please ensure that the statement adheres to our policy

The main data supporting the results in this study are available within the paper and its Supplementary Information. The raw and analysed datasets generated during the study are available for research purposes from the corresponding authors on reasonable request. Raw RNA sequencing data are available from the NCBI Gene Expression Omnibus under accession number GSE185433.

Human research participants

Policy information about studies involving human research participants and Sex and Gender in Research.

Reporting on sex and gender	For experiments with human monocytes, the sex of donors was unknown (anonymous donation). The volunteers for the human endotoxemia experiments were all male adults. The mice used for the biodistribution studies were female. The two non-human primates were male. Mice used for the tolerance-reversal model were all female.
Population characteristics	No female volunteers were included in the endotoxemia experiments. The ages of the male volunteers ranged from 18 to 35 years.
Recruitment	Volunteers for the human endotoxemia study were recruited via the distribution of flyers. No selection biases that might impact the results were expected by selecting the volunteers.
Ethics oversight	The endotoxemia model was approved by the CMO Arnhem-Nijmegen (Radboud University Medical Center), with registration numbers NL71293.091.19 and 2019-5730.

Note that full information on the approval of the study protocol must also be provided in the manuscript.

Field-specific reporting

Please select the one below that is the best fit for your research. If you are not sure, read the appropriate sections before making your selection.

☒ Life sciences ☐ Behavioural & social sciences ☐ Ecological, evolutionary & environmental sciences

For a reference copy of the document with all sections, see nature.com/documents/nr-reporting-summary-flat.pdf

Life sciences study design

All studies must disclose on these points even when the disclosure is negative.

Sample size	All in vitro experiments with primary human monocytes were performed in at least 6 biological replicates spread over at least 2 independent experiments. In our experience, these numbers provide sufficient power for the types of experiments performed in this study. In vivo experiments for biodistribution in mice were performed with 5 biological replicates; no statistical analysis was performed in the experiment, as we only visualized the biodistribution of the radiolabelled constructs. The biodistribution experiments in non-human primates were performed in two animals, owing to the exploratory nature of the experiment and to animal-ethics concerns. The human endotoxemia model was performed with in 8 individuals, owing to the limited availability of volunteers. The tolerance-reversal model was performed with n = 5 per experimental group (and 4 control mice); this number was based on a power calculation.
Data exclusions	No data were excluded.
Replication	All in vitro experiments with cells were performed independently at least twice, with the same outcome across experiments. Molecular biology experiments were replicated in three independent experiments, with similar results as presented in the paper. All data points for the in vivo experiments are individual mice or non-human primates.
Randomization	Animals for the in vivo experiments were randomly allocated to treatments groups when applicable. Primary human monocyte experiments and human endotoxemia experiments were paired in nature (comparing 'treated' to 'control' within the same individual), thus not requiring the randomization of individuals over treatment groups.
Blinding	Experiments with primary human monocytes were not blinded, since the nature of the measurements performed (and the objective outcomes) did not warrant blinding. RNA-sequencing data analysis was performed by an independent researcher using unbiased approaches. Comparisons of treatment with control in the same individual were not blinded. The animal experiments were not blinded during the conduction of the experiments for practical reasons, but the researcher performing serum ELISA was blinded.

Reporting for specific materials, systems and methods

We require information from authors about some types of materials, experimental systems and methods used in many studies. Here, indicate whether each material, system or method listed is relevant to your study. If you are not sure if a list item applies to your research, read the appropriate section before selecting a response.

Materials & experimental systems

n/a	Involved in the study
☐	☒ Antibodies
☐	☒ Eukaryotic cell lines
☒	☐ Palaeontology and archaeology
☐	☒ Animals and other organisms
☒	☐ Clinical data
☒	☐ Dual use research of concern

Methods

n/a	Involved in the study
☒	☐ ChIP-seq
☐	☒ Flow cytometry
☒	☐ MRI-based neuroimaging

Antibodies

Antibodies used	This information is provided in Methods. Specifically, flow-cytometry antibodies are described in Supplementary tables 3 and 5. Other antibodies are described in Methods for the assay they were used in.
Validation	All antibodies were commercially obtained and have been verified by the manufacturers. Verification data can be found by searching the manufacturers' website(s) using the provided catalog numbers. All antibodies were validated according to the manufacturers' websites using suggested working dilutions as described in their Technical Bulletins.

Eukaryotic cell lines

Policy information about cell lines and Sex and Gender in Research

Cell line source(s)	The HEK-Blue IL4/IL13 reporter cell line and HEK-293F were purchased from Invivogen.
Authentication	The HEK-Blue IL4/IL13 reporter cell line and HEK-293F were validated by the manufacturer. We did not perform additional authentication.
Mycoplasma contamination	The HEK-Blue IL4/IL13 reporter cell line and HEK-293F were delivered mycoplasma-free. After thawing, the cells were used immediately for the reporter assay or viral production, as described, and discarded afterwards. They were not kept in prolonged culture.
Commonly misidentified lines (See ICLAC register)	No commonly misidentified cell lines were used.

Animals and other research organisms

Policy information about studies involving animals; ARRIVE guidelines recommended for reporting animal research, and Sex and Gender in Research

Laboratory animals	Female C57BL/6J mice (approximately 8–11 weeks old and approximately 20 g) were purchased from The Jackson Laboratory. For non-human primate studies, two male cynomolgus monkeys (Macaca fascicularis) were used. All animals were co-housed in climate-controlled conditions at ambient temperatures (20–24 °C) and at 45–65 % humidity with 12-hour-light-dark cycles and provided water ad libitum. Mice were fed a standard chow diet, and the non-human primates were fed Teklad Global 20 % Protein Primate Diet.
Wild animals	The study did not involve wild animals.
Reporting on sex	All mice were female. However, the non-human primates were male, and so were the human volunteers for the endotoxemia experiments.
Field-collected samples	The study did not involve samples collected from the field.
Ethics oversight	Animal care and experimental procedures were based on approved institutional protocols from the Mount Sinai Institutional Animal Care and Use Committee (IACUC, Icahn School of Medicine at Mount Sinai) or the Nijmegen Animal Experiments Committee (Radboud University Medical Center).

Note that full information on the approval of the study protocol must also be provided in the manuscript.

Flow Cytometry

Plots

Confirm that:

☒ The axis labels state the marker and fluorochrome used (e.g. CD4-FITC).

☒ The axis scales are clearly visible. Include numbers along axes only for bottom left plot of group (a 'group' is an analysis of identical markers).

☒ All plots are contour plots with outliers or pseudocolor plots.

☒ A numerical value for number of cells or percentage (with statistics) is provided.

Methodology

Sample preparation

This information is provided in detail in Methods.

Macrophage surface-marker flow cytometry
Macrophages were harvested as described above and transferred to a v-bottom 96 well plate for staining. The cells were centrifuged at 1,500 rpm, 5 minutes, 4 °C. The supernatant was removed and the cells were washed once with 200 µl PBA (PBS pH 7.4, 1% w/v BSA (Sigma)).
Fc-receptors were blocked by incubation in PBS supplemented with 10% human pooled serum for 15 minutes at 4 °C. After washing once more, surface markers and viability were stained for in a volume of 50 µl for 30 minutes at 4 °C, using the antibodies and viability dye described in table 3. Following two washes, the cells were resuspended in 150 µl PBA and measured on a Cytoflex flow cytometer (Beckman Coulter) or BD FACSVerse system (BD Biosciences).

Phagocytosis assay
Macrophages were harvested as described above and incubated at 37 °C for 1 hour with FITC-labeled Candida albicans (kindly provided by Dr. Martin Jaeger, Radboudumc) at an MOI of 1:5. The cells were washed 2 times with ice-cold PBA and kept on ice to halt the phagocytosis. The cells were stained for CD45 (table 3) during 30 minutes in the dark at 4 °C. Following two washes, trypan blue was added to a final concentration of 0.01% to quench extracellular FITC-Candida. The cells were then acquired on a Cytoflex flow cytometer.

Cellular-specificity experiments
For cellular specificity, mice were intravenously injected with DiO labeled IL4-nanobiologics that was allowed to circulate for 24 hours. Subsequently, mice were sacrificed, and single cell suspensions were created from blood, spleen and bone marrow as previously described. Cell suspensions were incubated with anti-CD115, anti-CD11b, anti-Ly6C, anti-Ly6G, anti-CD19, anti-CD45, anti-CD11c, anti-CD3, anti-F4/80. Live/Dead Aqua was used as viability stain. Cells were subsequently washed and resuspended in FACS-buffer. All data were acquired on an Aurora 5L flow cytometer (Cytek Biosciences). DiO-IL4-nanobiologics were detected in the FITC channel.

T-cell polarization readout
In some experiments, harvested macrophages were used for subsequent T-cell-polarization assays. Allogeneic naïve T cells were seeded with macrophages in a ratio of 10 T cells for every macrophage. The cells were cultured in flat-bottom 96 well plates for 7 days in standard cell culture medium. In this model, HLA mismatch causes non-specific activation of the T cell receptor. On the final day, the cells were stimulated with PMA (25 ng/mL) + ionomycin (0.5 µg/mL) for 4 hours in the presence of 100 ng/mL Brefeldin A, a 'golgi-plug'. The cells were harvested and split over 2 flow-cytometry antibody panels (one for CD4 T cells and one for CD8; see also table 3). The cells were stained in a similar manner as described above, with an extra step for permeabilization of the T cells to allow for intracellular cytokine staining. This was performed using the Fix/Perm buffer set (eBioscience), according to the manufacturer's instructions. The gating strategy was similar to what is described above, with the addition of a selection for CD3-positive events. The percentage of cells positive for hallmark cytokines of T cell polarization were calculated to estimate T cell subset proportions.

Phospho-STAT6 measurement by flow cytometry
Monocytes were stimulated with RPMI, IL4, or different concentrations of IL4-aNPs (indicated in the figure) for 20 minutes at 37 °C. The cells were transferred to a v-bottom 96 wells plate and kept on ice for the duration of the staining procedure. After staining for viability and CD14 (in the manner described above), the cells were fixed and permeabilized using the fix/perm buffer set (eBioscience) for 45 minutes at 4 °C in the dark. The cells were washed twice with perm buffer and incubated overnight in freezer-chilled absolute methanol at -20 °C overnight. Following two more washes in perm buffer the cells were stained for phospho-STAT6 using the antibody described in table 3, for 45 minutes at 4 °C in the dark. The cells were washed two more times in perm buffer and finally resuspended in PBA for acquisition on the Cytoflex cytometer. The gating strategy was largely similar to the one for macrophage surface marker with the addition of a selection for CD14-positive events.

Instrument

BD FACSVerse / Beckman Coulter Cytoflex / Cytek Biosciences Aurora 5L

Software

Flowjo (v10.7.1, BD Biosciences) and Graphpad Prism (V9, Graphpad Software).

Cell population abundance

The starting material purity of the primary human monocytes was assessed using a Sysmex automated hemocytometer.

Gating strategy

Gating strategies are detailed in Methods. Briefly: data analysis was performed in Flowjo (v10.7.1, BD Biosciences). Our gating strategy was as follows: first, a time gate was used if necessary. Then, single-cell events were selected using subsequent FSC-A/SSC-A and FSC-A/FSC-H gates. Dead cells were removed from the analysis by selecting the viability dye-negative population. Geometric mean fluorescence intensities were calculated as a measure of macrophage surface-marker

expression in trained-immunity experiments. For phagocytosis experiments, CD45+ events were first selected to remove Candida-only events. The percentage of Candida-FITC positive macrophages is reported. For the cellular-specificity (biodistribution) assay, the percentage of IL4NB-DiO positive cells are reported.

☒ Tick this box to confirm that a figure exemplifying the gating strategy is provided in the Supplementary Information.
